# Regulatory elements coordinating initiation of chromosome replication to the *Escherichia coli* cell cycle

**DOI:** 10.1073/pnas.2213795120

**Published:** 2023-05-23

**Authors:** Anna Knöppel, Oscar Broström, Konrad Gras, Johan Elf, David Fange

**Affiliations:** ^a^Department of Cell and Molecular Biology, Science for Life Laboratory, Uppsala University, Uppsala 75124, Sweden

**Keywords:** DNA replication initiation, cell growth, *Escherichia coli*, DnaA

## Abstract

*Escherichia coli* coordinates replication and division cycles by initiating replication at a narrow range of cell sizes. By tracking replisomes in individual cells through thousands of division cycles in wild-type and mutant strains, we were able to compare the relative importance of previously described control systems. We found that accurate triggering of initiation does not require synthesis of new DnaA. The initiation size increased only marginally as DnaA was diluted by growth after *dnaA* expression had been turned off. This suggests that the conversion of DnaA between its active ATP- and inactive ADP-bound states is more important for initiation size control than the total free concentration of DnaA. In addition, we found that the known ATP/ADP converters *DARS* and *datA* compensate for each other, although the removal of them makes the initiation size more sensitive to the concentration of DnaA. Only disruption of the regulatory inactivation of DnaA mechanism had a radical impact on replication initiation. This result was corroborated by the finding that termination of one round of replication correlates with the next initiation at intermediate growth rates, as would be the case if RIDA-mediated conversion from DnaA-ATP to DnaA-ADP abruptly stops at termination and DnaA-ATP starts accumulating.

## Significance

Bacteria replicate the genome on average once per generation, irrespective of growth condition–dependent changes in generation time and cell size. The strategy appears to be to initiate replication in a narrow chromosome-to-size ratio interval for each growth condition. To investigate how *Escherichia coli* accurately compares its size to the number of chromosomes, we perturbed some of the mechanisms proposed to achieve this precision and monitored how replication initiation was affected with time-lapse single-cell fluorescence microscopy. We found that the regulatory inactivation of DnaA (RIDA) mechanism, which converts the replication initiation molecule DnaA to its inactive form in proportion to the number of chromosomes, was the most important factor and that other previously described mechanisms have a less critical role.

All cells need to coordinate replication with cell growth such that each chromosome is, on average, replicated once per generation. This is particularly challenging for bacteria with a wide range of generation times, some even shorter than the time it takes to replicate the chromosome (1). The ambition of this work is to understand how *Escherichia coli *coordinates replication initiation with its biomass production in order to achieve the narrow cell size distribution at initiation that was recently observed at the single-cell level (2, 3, 4, 5).

More than 50 y ago, Donachie (6) noted that the media-dependent cell size observed by Schaechter et al. (7) could be explained if *i)* initiation of replication occurs at the same ratio of cell size and number of origins of replication in different media and *ii)* division occurs at a constant and media-independent time from initiation [as suggested by Cooper and Helmstetter (8)]. The question regarding the constancy of the cell size-to-origin ratio at initiation is still an active field of research, with recent publications arguing both for (3, 9) and against (10).

The essential protein DnaA is generally described as a key contributor in triggering initiation of chromosome replication in *E. coli* (11). DnaA bound to ATP can initiate replication in vitro (12), and the intracellular concentration of DnaA has been shown to negatively correlate with cell size at initiation (3, 13, 14, 15). There are, however, also reports of the initiation size being insensitive to moderate (≤ twofold) changes in the intracellular DnaA concentration (16). In addition to DnaA concentration, several mechanisms that contribute to initiation regulation have been described, including, e.g., the autoregulation of *dnaA* expression (17), SeqA-dependent sequestration of newly replicated *oriC* to prevent reinitiation (18), and binding of DnaA-ATP (the active form of DnaA in replication initiation) to DnaA boxes throughout the chromosome (19). A number of systems have been found to modulate DnaA’s state of ATP or ADP binding. The chromosomal locus *datA* and the replication machinery–associated Hda protein both reduce the initiation potential by promoting the hydrolysis of DnaA-ATP to DnaA-ADP (20, 21, 22, 23, 24). Conversely, the chromosomal loci *DARS1* and *DARS2* (25, 26), and membrane-associated acidic phospholipids (27), have been described to convert DnaA-ADP to the apo-form. The apo-form then quickly binds primarily ATP, as the cellular concentration of ATP is higher than the concentration of ADP, and DnaA has the same affinity for both nucleotides (28, 29). In addition, several auxiliary proteins that can promote the function of some of the above mechanisms have been described. DiaA has been suggested to assist in the oligomerization of DnaA on the origin (30). IHF increases DnaA affinity to parts of the *oriC* region (31) and is required for *datA* (21) and *DARS2* (32) function. Additionally, Fis is required for *DARS2* function (32, 33). It is, however, still not known how these systems operate together and if they are sufficient to regulate replication initiation such that it keeps up with biomass production.

In recent years, the genetic insights have been complemented by a more phenomenological path to study initiation control. The path emerged from breakthroughs in live-bacterial imaging and the invention of the mother machine microfluidic chip (34). This path initially focused on the statistical properties of cell size variation from generation to generation since this was what could be measured with phase-contrast microscopy alone (35, 36). It was, however, clear that these studies could only define limits that mechanistic models should satisfy (35, 36, 37). Mechanistic insight can however be gained by using fluorescence microscopy. This had previously been done using low-throughput methods (38, 39, 40). However, more recently, high-throughput observations of ongoing replication at the single-cell level have been made (2, 3, 4, 9, 41, 42), where the Boesen et al. preprint appeared during the review process of the current paper (42).

By tracking replisomes for multiple generations, Si et al. (3) showed that the cell size at initiation of replication correlates with the cell size at the previous initiation in such a way that the added size increment between the two initiations is, on average, constant and independent of the cell size at the first initiation. This *initiation adder* was previously suggested based on model analysis (43, 44, 45) and was corroborated experimentally by Witz et al. and Colin et al. (4, 5). An adder phenotype can, for example, arise if the cell accumulates an initiator molecule proportionally to its volume and initiates replication when it has accumulated a fixed number of initiator molecules per origin (3, 43). DnaA was implicated as the accumulating initiator molecule, but the mechanistic details of if and how DnaA can accomplish this are still unknown.

In this study, we have studied replication and division cycles using high-throughput fluorescence microscopy for a number of genetically perturbed strains in order to gain insights into the importance of each component. We began by characterizing the coordination of replication with the division cycle at different growth rates, then assessed the importance of DnaA regulation, expression, and abundance, and finally examined the relative contributions of the various systems modulating the DnaA-ATP and -ADP bound states. The results are evaluated in relation to previously described models for replication initiation control (Box 1).

Box 1. Possible mechanisms for balancing replication initiation with cell growth

**Box 1.**
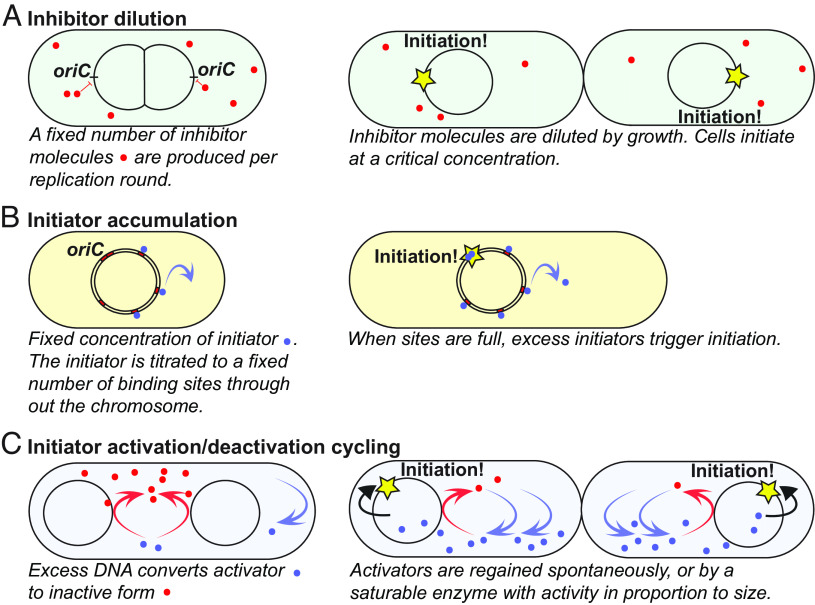
Models for coordination between growth and replication initiation. (*A*) Inhibitor dilution model. (*B*) Initiation accumulation model. (*C*) Initiator acativation/deactivation cycling.

On average, cells trigger a new round of replication on each origin once per generation. The cell size at replication initiation has little variation in a constant medium (46). Principally, different mechanisms for achieving this level of regulation have previously been proposed. The key elements of these different mechanisms are introduced here. The actual implementations used by the cells will not be as clear-cut and can obviously include components from different mechanisms, for example, as in the models by Berger and Wolde and Grant et al. (47, 48).

***A. Inhibitor dilution****:* Each copy of the genome expresses a constant number of replication inhibitors per genome per generation. The inhibitors are diluted by growth such that initiation is triggered when the inhibitor concentration decreases to a specific value (49, 50, 51, 52). This does not give a sensitive response in inhibitor concentration to volume ratio and it would need to be complemented with a sensitive response regulator at the *oriC* (53). In its purest form, the mechanism would be a sizer with respect to cell size at initiation.

***B. Initiator accumulation****:* The initiator is kept at a constant concentration in the cell such that the number of molecules is proportional to the cell size (54, 55). The initiators bind strongly to a fixed number of sites per chromosome (55, 56). When these sites are full, excess synthesis contributes to increasing the concentration of free initiators, and thus, initiation is triggered at a fixed volume per chromosome each generation. This mechanism results in an adder phenotype with respect to initiation volume.

***C. Initiator activation/deactivation cycling****:* The initiator is cycled between an active and an inactive state, where the flux to the active state increases with increasing volume and the flux to the inactive state increases with the number of chromosomes. If the modification reactions are saturable, a mechanism triggering at a fixed chromosome-to-volume ratio can be made very sensitive to the balance of fluxes (53, 57). If the fluxes have no memory, the mechanism would be a sizer with respect to initiation volume.

***D. Mechanical models*** *(not in the figure):* There is also a class of initiation models where replication is triggered based on other processes in the cell cycle, effectively shifting the regulatory problem to these processes. For example, if replication is triggered after cell division (40), the challenge is not to initiate at a set size, but to divide at a set size. If replication is triggered by termination, the challenge is to adapt the replication rate to the growth rate.

## Results

### Experimental Setup, Labeling, and Interpretation.

**Fig. 1.**
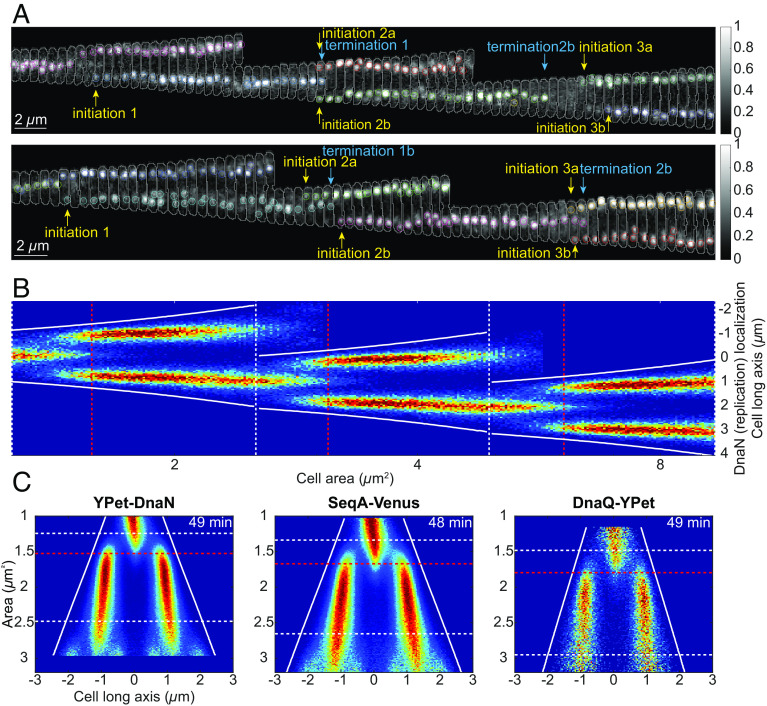
Experimental setup and data visualization. (*A*) Examples of cells tracked over three generations. Detected YPet-DnaN foci are marked with circles, where circles sharing the same color indicate replication started from the same origin, i.e., two replication forks moving bidirectionally on the chromosome, and are often spatially colocalized. The foci were tracked using the u-track algorithm (61) (*Materials and Methods*). Initiations (marked with yellow arrows) are defined as the start of u-track tracks, and terminations (marked with blue arrows) are the end of u-track tracks. Cell outlines are displayed with a gray line. (*B*) Three-generation fork plot. A two-dimensional distribution of the position of YPet-DnaN foci along the long axis of the cells (*y* axis) and cell area, from here on referred to as cell size (*x* axis), is plotted. Here, the cell size in generations two and three is defined as if the cells in the previous generation had not divided. We refer to this type of structure as a “supercell.” Dashed white lines indicate the average sizes of cells at birth or division. Dashed red lines indicate the average initiation size as determined by tracking replisomes in single cells. Solid white lines indicate average cell pole positions. (*C*) One-generation fork plots for three different replisome markers. Here, the *x* axis shows the cell’s long axis and the *y* axis shows cell size given in µm^2^. Doubling time is given in the top right corner of each plot. Lines as in *B*. Statistics for *B* and *C* can be found in Dataset S1.

In order to study replication in relation to the cell cycle, we grew *E. coli* MG1655 cells in a mother machine–type fluidic chip that allowed for rapid medium swaps (58). We monitored thousands of individual cell cycles in each experiment. Growth was imaged by phase-contrast, and replication forks were localized by detecting the fluorescent foci (Fig. 1*A*) of YFP translational fusions to DnaN, DnaQ, or SeqA. DnaN and DnaQ are both parts of the replisome (59), and SeqA binds hemimethylated GATC in the wake of the complex (18). Experiments with the different replication markers (Fig. 1*C*), or at different temperatures (*SI Appendix*, Fig. S1*A*), produced very similar results. The localization of replication forks in thousands of cells sorted on size is presented in the so-called fork plots, which are either plotted for multiple generations (Fig. 1*B*) or one generation (Fig. 1*C*) (60). We refer to the densest regions of the fork plots as branches. The start of each branch is localized in the same long-axis position as *oriC* (*SI Appendix*, Fig. S1*B*) and is interpreted as the initiation of individual rounds of replication, with two replication forks moving bidirectionally away from the origin region (2).

### Spatial Organization of Replication Is Not Dependent on Growth Condition or Cell Division.

**Fig. 2.**
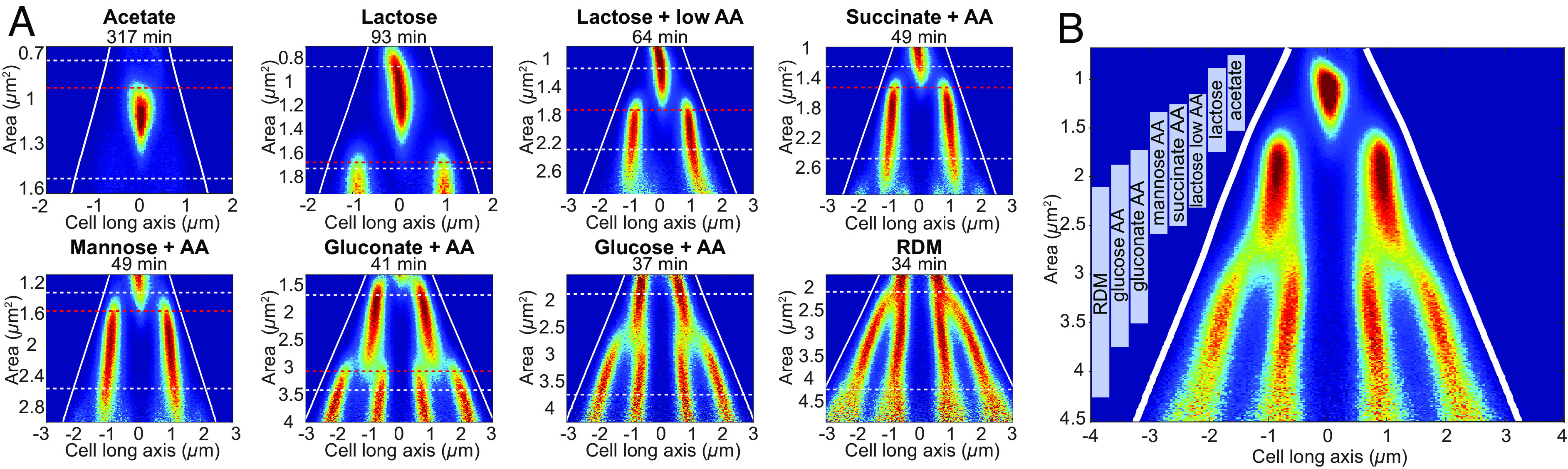
Spatial organization of the replication machinery in different growth media. (*A*) Fork plots for strains carrying YPet-DnaN grown in different media (*SI Appendix*, Table S1). The medium is specified above each fork plot, and the corresponding doubling time is found above each plot. Lines as in Fig. 1*B*. (*B*) Pooled fork plot with the data from *A* with the same number of cells from each growth condition. Vertical bars indicate the division cycle of each medium. Statistics can be found in Dataset S1.

*E. coli* growing in different media have varying degrees of overlapping replication and divide at different sizes (7, 8). Therefore, it could be possible that replication has different spatial organization under different growth conditions. To quantify the effect of the growth medium on replisome organization, we imaged YPet-DnaN-expressing cells (with *dnaA* expressed from its native promoter) in eight different growth media (Fig. 2*A*). We found that the cells appeared to use the same guiding principle to spatially organize their replication regardless of the growth medium, although the number of concurrent replication processes differed. On average, cells grown in acetate initiated replication at one origin; cells grown in lactose [with and without amino acids (AA)], succinate + AA, and mannose + AA concurrently initiated replication at two origins; and cells grown in gluconate + AA, glucose + AA, and rich defined medium (RDM) initiated replication at four origins. To highlight the similarities in replication organization, we pooled the data from all different growth media and constructed a fork plot for the entire set in a common coordinate system (Fig. 2*B*). Apart from cells grown in RDM, the cells initiated replication at a similar ratio of cell size to the number of concurrently initiated origins (*SI Appendix*, Table S2), giving the pooled fork plot a well-groomed appearance (Fig. 2*B*). The pooled representation fits nicely with previous observations where the average division sizes can be perturbed without affecting the initiation sizes (15). We will refer to the ratio of cell size at initiation and the number of origins on which replication concurrently occurs as the initiation size-to-origins ratio. The cell areas (from now on sizes) at division were found to increase by 2.8-fold, partly due to an increase in cell width, when going from acetate to RDM media (Fig. 2*A* and *SI Appendix*, Table S2), which, in turn, required the use of differently sized microfluidic growth chambers to keep the cells well organized for imaging. The average initiation size-to-origins ratio was, for cases where we could detect initiation events, found to decrease by 17% (*SI Appendix*, Table S2) as the doubling time decreased from 317 min (acetate) to 41 min (gluconate AA). The trend should be interpreted with some caution, since comparing cells of very different sizes could introduce biases in the size estimations due to differences caused by how the cells fill microfluidic channels.

In line with previous observations that replication termination affects division only when they occur at similar sizes (4) and that the replication and division processes are uncoupled (3), we found that correlations between initiation-subsequent division sizes and termination-subsequent division sizes disappear as the growth rate increases (*SI Appendix*, Table S3). Even more important for this investigation, however, is that since initiation can occur right before division (e.g., for cells grown in gluconate + AA) or right after division (e.g., for cells grown in mannose + AA), division appears unimportant for triggering replication at intermediate growth rates.

### Initiation of Replication Can Be Accurately Triggered without Autoregulated Expression of the *dnaA Gene*.

When we tracked replication forks at the level of single cells, we observed correlation coefficients of 0.3 to 0.5 between the cell size of two subsequent replication initiation events, independently of growth conditions (*SI Appendix*, Fig. S2 and Table S3). This is in line with previous observations (3, 4, 5) and is incompatible with a sole cell size sensor–triggering initiation. For exponentially growing cells, a correlation coefficient of 0.5 is typical of an adder phenotype where an, on average, constant and independent cell size increment is added between two subsequent divisions or initiations [the correlation coefficient is derived for subsequent divisions in the studies by Amir (37) and Taheri-Araghi et al. (36), but applies equally well to initiations]. The adder phenotype can for example arise if the cell accumulates an initiator molecule proportionally to its volume and initiates replication when it has accumulated a fixed number of initiator molecules per origin (3, 43). This corresponds to the initiator accumulation model in Box 1. The natural candidate for the initiator is DnaA; DnaA bound to ATP can initiate replication in vitro (12) and the intracellular concentration of DnaA has been shown to negatively correlate with cell size at initiation (3, 13). Additionally, DnaA-ATP is known to autoregulate its own transcription (62), which would be favorable in a situation where the free concentration of DnaA-ATP should be kept constant and the number of free molecules proportional to the cell size.

**Fig. 3.**
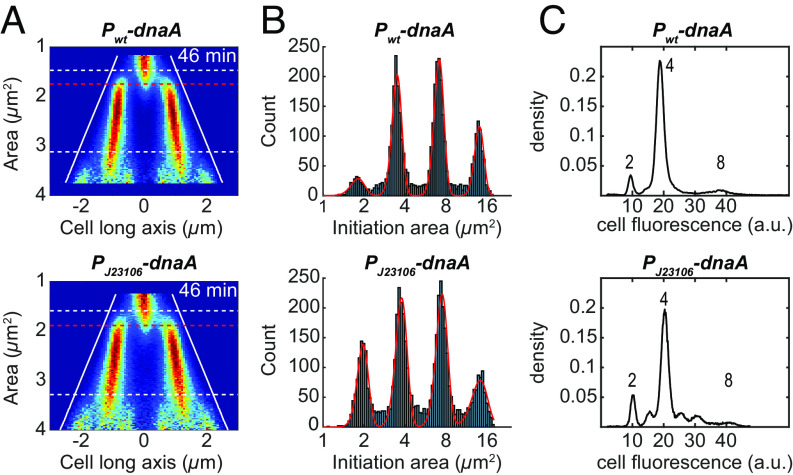
Replication initiation phenotypes for constitutive DnaA expression. (*A*) Fork plots for SeqA-Venus in strains carrying either the native promoter of *dnaA* (*P*_*wt*_*-dnaA*), or a constitutive promoter with a similar DnaA expression level (*P*_*J23106*_*-dnaA*). The cells were grown in succinate + AA medium at 30 °C. Lines as in Fig. 1*B*. (*B*) Distributions of initiation sizes from cells shown in *A* that have been tracked for at least four generations. The initiation size distribution is fitted with a sum of four Gaussians. Initiation sizes are here given as if the cells had not divided. (*C*) Distributions of DNA-stain fluorescence in single cells captured using flow cytometry following a rif-runout experiment (14). The cells have different promoters for *dnaA*, but no fluorescent markers on the replisomes. The absolute magnitude of the number of origins (shown above each peak) has been inferred from the fork plots in *A*. For methods in *C*, see *SI Appendix*, *SI Materials and Methods*. Statistics for *A* and *B* can be found in Dataset S1.

To investigate whether autoregulation of *dnaA* transcription is important for upholding the well-defined initiation size distribution, we constructed a strain in which the expression of a sole copy of *dnaA* is constitutive. This was done by introducing *dnaA* under the control of a constitutive promoter (J23106) at a separate position of the chromosome and by exchanging the two native promoters of the *dnaA* operon as well as the *dnaA* gene with a constitutive promoter (J23106) in front of the remainder of the operon (*dnaN* and *recF*). The *P_J23106_-dnaA* strain showed similar average expression of DnaA (*SI Appendix*, Table S4), but increased expression of DnaN and RecF (2.8 and 2.3-fold, respectively; *SI Appendix*, Table S4) as compared to the *P_wt_*-*dnaA* strain. The *P_J23106_-dnaA* strain also has similar phenotypes as the *P_wt_*-*dnaA* strain (Fig. 3 *A* and *B*), with a 4% increase in average initiation size, a 12% increase in average division size, as well as a 13% increase in the coefficient of variation (CV) for the distribution of initiation sizes (*SI Appendix*, Table S4). In addition, the *P_J23106_-dnaA* strain also showed a small increase in replication asynchrony (Fig. 3*C*), and it displayed an indistinguishable growth rate compared to the *P_wt_-dnaA* strain, both when grown in the microfluidic chip and in a plate reader (*SI Appendix*, Table S4). We concluded that DnaA’s ability to control its own expression does not have a critical role in the regulation of replication initiation. For the remainder of the paper, we primarily use strains without autoregulation of *dnaA* expression, since this often makes interpretations less ambiguous.

### Active Transcription of the *dnaA* Gene Is Not Needed to Trigger Replication Initiation.

In a version of the initiator accumulation model where gene expression supplies the initiator molecule [as suggested in ref. (43)] and where DnaA is the initiator molecule that is being accumulated [as suggested in refs. (3, 42)], new synthesis of DnaA is needed for replication initiation. In this model, shutting off *dnaA* expression would prohibit further initiations.

To test this prediction, we used a strain where the sole copy of the *dnaA* gene is placed behind the isopropyl β-D-1-thiogalactopyranoside (IPTG) controllable *lac* promoter (*P_lac_-dnaA* strain). RT-qPCR measurements of the *dnaA* transcript before and after removing 1 mM IPTG (corresponding to maximal induction) showed that *dnaA* was effectively repressed when IPTG was removed (*SI Appendix*, Fig. S3*A*). Using OD measurements, we also found that cells could grow at an unperturbed rate for about six generations following *dnaA* repression and eventually, the removal of IPTG led to growth arrest (*SI Appendix*, Fig. S3*B*). Under the microscope, we eventually observed replication initiation arrest and the presence of filamentous cells after 1 mM IPTG removal (Movie S1). To distinguish whether cells stopped initiating replication due to the lack of expression of *dnaA* or due to its low intracellular concentration, we supplied DnaA in excess (using 1 mM IPTG) before turning off its gene expression. In this way, we had time to observe possible initiations before the DnaA concentration became too low.

**Fig. 4.**
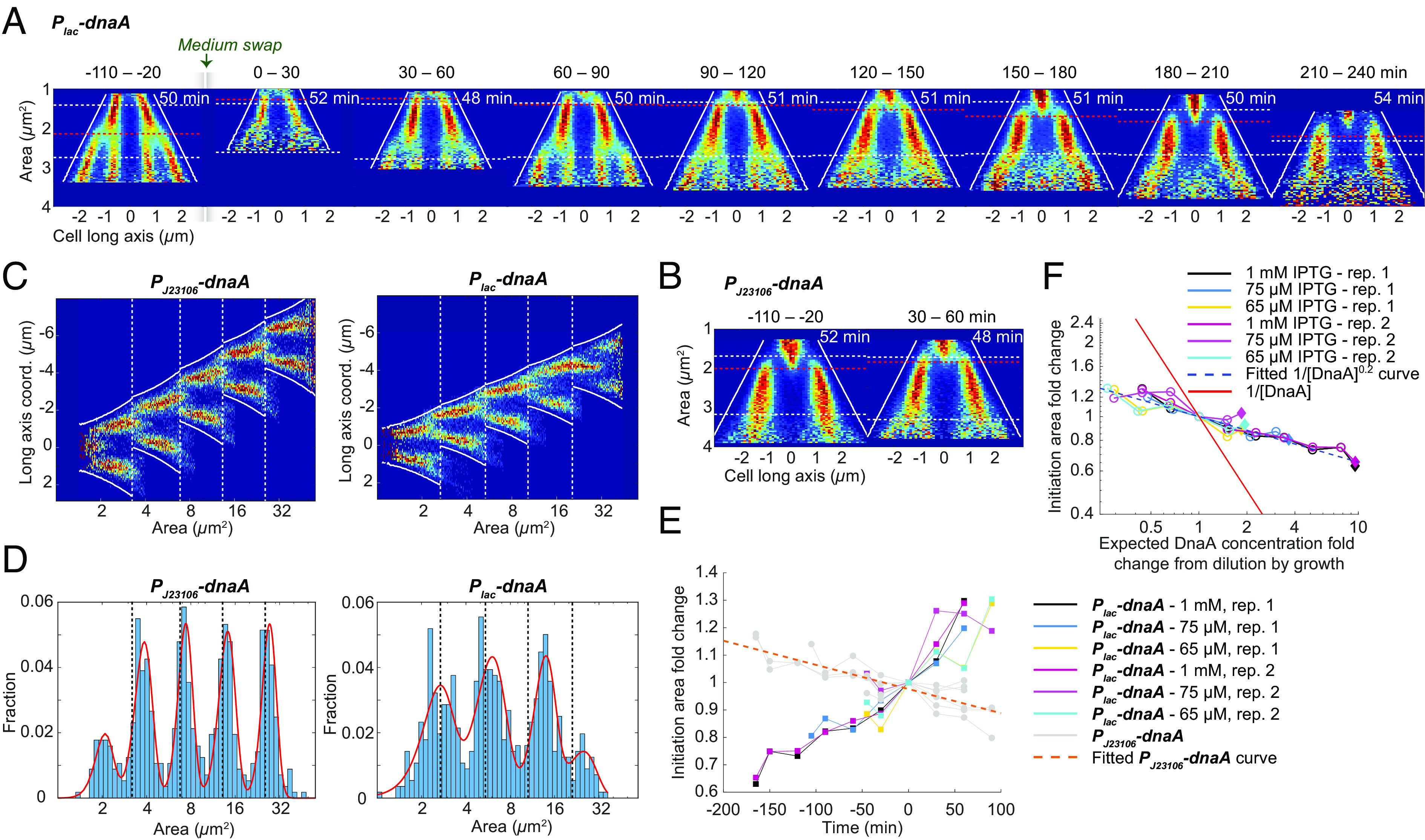
Replication initiation continues after turning off the expression of *dnaA*. (*A*) Fork plots for a strain with IPTG-controllable DnaA expression (*P*_*lac*_*-dnaA*) before (−110 to −20 min) and after (0 to 240 min) the medium was swapped from medium with 1 mM IPTG to medium without IPTG. Lines and numbers as in Fig. 1*B*. (*B*) As in *A* but for a strain with constitutive expression of DnaA (*P*_*J23106*_*-dnaA*) grown in the same microfluidic chip. One representative fork plot after the medium swap (30 to 60 min) is shown. (*C*) Five-generation fork plot after medium swap (0 to 240 min) for constitutive (*Left*) and IPTG-regulated (*Right*) *dnaA* expression. Cells in consecutive generations were part of the same lineage and located at the bottom of the microfluidic channels. (*D*) Distribution of single-cell initiation sizes (blue bars) of the same cells as in *C*. Red lines show the result of a regression of the distribution to either a sum of five (*Left*) or a sum of four (*Right*) Gaussians. Dashed black lines indicate cell division. (*E*) Fold change in initiation sizes over time for six *dnaA* turn-off experiments with different initial expression levels. Time 0 and fold change 1 are defined as where the replication phenotype of the *P*_*lac*_*-dnaA* strain and the reference *P*_*J23106*_*-dnaA* strain was the most similar. The orange line corresponds to a linear fit for all *P*_*J23106*_*-dnaA* replicates in the *dnaA* turn-off experiments. (*F*) Fold change of initiation size against expected DnaA concentration by growth dilution after swap to medium without IPTG. Here, 1 is set to the initiation size that corresponds to the reference strain carrying *P*_*J23106*_*-dnaA*. The red curve corresponds to $\frac{1}{\left[DnaA\right]}$ . The dashed blue curve corresponds to a fit of all data points to $\frac{1}{{\left[DnaA\right]}^{a}}$ with the exponent a = 0.2. Filled diamonds indicate data before the removal of IPTG and open circles indicate data after the removal of IPTG. Statistics can be found in Dataset S1.

After turning off DnaA synthesis, cells gradually initiated replication at larger and larger initiation size-to-origins ratio as the DnaA concentration decreased by dilution (Fig. 4*A*). 160 minutes, or ~3 generations, after DnaA synthesis was turned off, the initiation size in the *P_lac_-dnaA* strain was the same as for the *P_J23106_-dnaA* strain (Fig. 4 *B* and *E*). Within the next generation following this point, the low DnaA concentration could not sustain regular initiations (Fig. 4). For further verification, we tracked only the cells at the constricted end of the microfluidic channels for five generations after the medium swap and saw that they were able to repeatedly initiate replication in the same manner as the whole population (Fig. 4 *C* and *D*). We also turned off DnaA synthesis starting from two lower DnaA expression rates (induced by 75 µM and 65 µM IPTG). We found that the time it takes to reach the initiation size of the *P_J23106_*-strain depends on the initiation size before turning off DnaA expression (Fig. 4*E*), where a larger initial initiation size gives a shorter time. Additionally, we found that if the data are aligned by the time at which the inducible strain has the same phenotype as the *P_J23106_*-strain, cells started at different *dnaA* expression levels follow the same path in the reduction of initiation sizes with only the starting point differing (*SI Appendix*, Fig. S3*C*). Using lower IPTG concentrations (65 µM and 75 µM) could potentially introduce heterogeneity in the expression rate of DnaA. This will however not affect the statements about the average cell behaviors presented here.

For DnaA concentrations within a twofold change from wild type, recent reports have found an inverse proportionality between initiation size per origin and the intracellular DnaA concentration (42). As in many other studies before this [(42) and references therein], we have tried to directly quantify the intracellular DnaA concentration. However, due to nonconvergence and partly high experiment-to-experiment variability for the different methods tried (RT-qPCR, LC-MS/MS, and microscopy using fluorescence reporters), we found these results inconclusive. Instead, we rely on the fact that the change in DnaA concentration in two consecutive generations is reduced at least twofold after the expression of *dnaA* is turned off. We found that the increase in initiation size as a response to the reduction in DnaA concentration due to dilution by growth is weaker than expected from an inverse proportionality (Fig. 4*F*). The initiation size follows the curve 1/[DnaA]^0.2^ instead of the curve 1/[DnaA].

We concluded that the result from turning off *dnaA* expression from an overexpressed level contradicts models exclusively dependent on gene expression–based accumulation of DnaA to a threshold level where initiation is triggered. This would, for example, rule out the specific model where new synthesis of DnaA is titrated to binding sites that have higher affinity than that of *oriC*, allowing initiation to occur only when these sites are filled (i.e., Box 1*B*). Furthermore, since the chromosome concentration changed less than twofold when *dnaA* was significantly overexpressed (>eightfold) (Fig. 4*F*), excess DnaA alone cannot trigger initiation. The presence of separated peaks of initiation sizes in consecutive generations (Fig. 4*D*) after DnaA expression was turned off instead indicates that cells did not initiate replication directly after origin sequestration had ended, i.e., 15 min after initiation (63). This implies that excess DnaA is somehow prevented from initiating replication after origin sequestration, possibly due to the presence of an inhibitor or because DnaA has been converted into a nonactive form. DnaA-ADP could have been a plausible inhibitor of initiation, but, although DnaA-ADP cannot initiate replication by itself, it has been shown to promote rather than inhibit initiation when present together with DnaA-ATP (64, 65, 66). RIDA, on the contrary, is a well-documented example of a system that converts the active initiator form into the inactive (22), even in strains with high overexpression of DnaA (23). Direct measurements of DnaA-ADP and DnaA-ATP concentrations in bulk samples also suggest that DnaA-ADP dominates (23). It seems most likely that the reason excess DnaA does not trigger initiation is that most of the molecules are inactive.

### Deletion of the *hda* Gene Gives a Growth Rate–Dependent Initiation Phenotype.

**Fig. 5.**
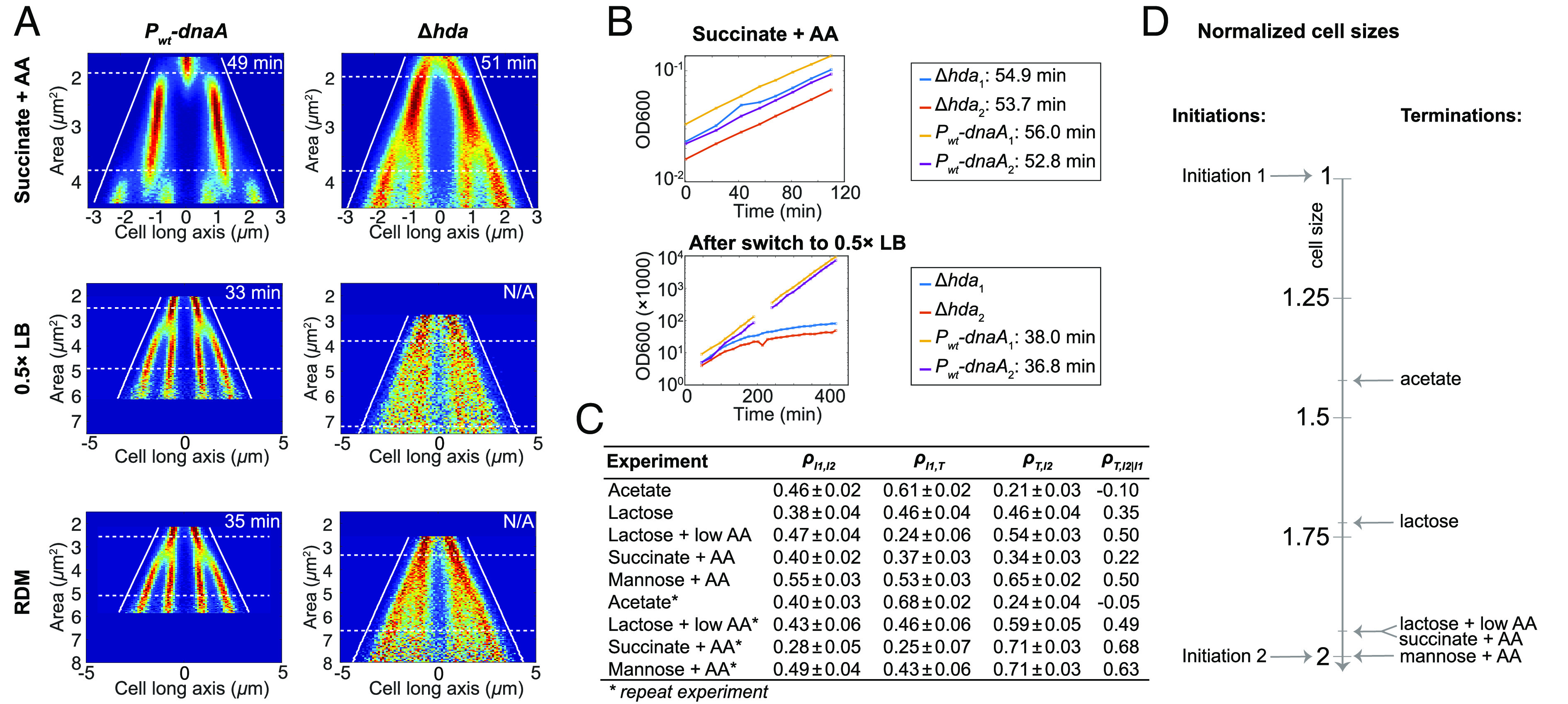
Replication phenotype of Δ*hda* and cell size correlation coefficients between initiation and termination events. (*A*) Fork plots of the Δ*hda* and *P*_*wt*_*-dnaA* strains grown in succinate + AA, 0.5× LB, and RDM media. For the cases of 0.5× LB and RDM, the cells were initially kept in succinate + AA medium and then swapped to either RDM or 0.5× LB. The fork plots shown are of cells imaged between 160 and 240 min after the medium swap. The full experiments are presented in *SI Appendix*, Fig. S4. (*B*) Batch growth of the same strains as in *A* grown first in succinate + AA medium then swapped to 0.5× LB. The *P*_*wt*_*-dnaA* cells were rediluted 50-fold at 192 min after swapping to 0.5× LB. For visualization and simplified growth rate estimation, the presented values after redilution are OD_600_ × 50 × 1000. (*C*) Cell size correlation coefficients (ρ) between events I_1_, I_2_, and T as defined in the main text from the YPet-DnaN strain grown in various media. ρ_T, I2 | I1_ is the partial correlation between T and I_2_ with the effect of I_1_ accounted for. The correlation coefficients are found by fitting a bivariate Gaussian function (*Materials and Methods*) to the two-dimensional histograms shown in *SI Appendix*, Fig. S3. ± indicates 67% CIs of the regression estimate. (*D*) Cartoon highlighting the cell size relations for initiation and termination in the different growth conditions used. Statistics for *A* and *C* can be found in Dataset S1.

The Hda protein has been shown to be necessary for RIDA functionality (24). To investigate the consequences of RIDA inactivation, we constructed a Δ*hda*::*kanR* mutant in a SeqA-Venus-labeled *P_wt_*-*dnaA* strain (*SI Appendix*, *SI Materials and Methods*). We used whole-genome sequencing to ensure that the *hda* deletion strain had not acquired any of the previously described compensatory mutations (67). While the division sizes in the *hda* deletion mutant were similar to those of the *P_wt_-dnaA* strain when grown in succinate + AA medium (Fig. 5*A*), the initiation size-to-origins ratio was substantially smaller than that of the *P_wt_-dnaA* strain, with initiation primarily occurring on four origins in the Δ*hda*-strain as compared to primarily on two origins in the *P_wt_-dnaA* strain. The branches in the fork plot for the *hda* deletion mutant also lack clearly defined starting points, suggesting high variability in the cell sizes at initiation. Similar results were observed in a recent preprint by Boesen et al. (42). The *hda* deletion-strain grew indistinguishably from the *P_wt_-dnaA* strain in succinate + AA medium, but when the medium was swapped from succinate + AA to either RDM or 0.5× LB, the initiation size became undefined (Fig. 5*A* and *SI Appendix*, Fig. S4) and the cell growth rate decreased gradually until growth stopped (Fig. 5*B*). A growth defect is also what we would expect if Hda was the main mechanism to convert DnaA to the ADP-form. A *hda* deletion would in this case result in significant overinitiation, where initiation is triggered more frequently than the time it takes to double the cell mass, causing a detrimental accumulation of DNA. That deletion of *hda* results in overinitiation is also in line with previous reports that overexpression of the origin sequestration protein SeqA can suppress loss of Hda (67).

RIDA is dependent on active replication (22). This implies that if there are competing processes converting between the ATP- and ADP-forms of DnaA, the net flux of DnaA-ATP production should increase following termination of replication and possibly trigger new initiations. To test whether there is such an effect, we recorded the cell size at two subsequent initiations, hereafter referred to as I_1_ and I_2_, together with the cell size at the termination (T) associated with I_1_, and correlated them with one another. To ensure that we could determine a meaningful termination-to-initiation size correlation, we first made sure that the ends of the time-lapse fluorescent foci trajectories were truly indicating termination events by showing that replisomes and termini (*ter*-mCherry; *SI Appendix*, Fig. S5 for fork plots with the different carbon sources) are close in space when the YPet-DnaN trajectories end (*SI Appendix*, Fig. S6).

Since we found correlations both between the cell size at I_1_ and I_2_ (Fig. 5*C*, left-most column) and between I_1_ and T (Fig. 5*C*, second column), we expect I_1_ to be a confounding variable for the correlation between T and I_2_, potentially giving rise to spurious correlations. To address this issue, we used partial correlations (68) between T and I_2_ in which the effect of I_1_ on both T and I_2_ is accounted for. Intuitively, we expect the correlation between T and I_2_ to be very small for cells grown in acetate, since the termination (T) and subsequent initiation (I_2_) occur about half a cell cycle apart (Figs. 2 and 5*D*). This is also what we found for cells grown in acetate, where the partial correlation between T and I_2_ is ~0 (Fig. 5*C*). For cells grown in media where termination and initiation occur at similar cell sizes (Fig. 5*D*), the correlation is, however, higher. For example, the cells grown in mannose + AA have a partial correlation coefficient between T and I_2_ of about 0.5. Similar results (except for acetate) (69) appeared during the review process of the current paper. The observed increase in correlation between termination and initiation could be explained by the cessation of RIDA at termination. Note that the correlation analysis was not performed in faster growth conditions with overlapping replication cycles (see marker frequency measurements in *SI Appendix*, Fig. S7) as it is difficult to accurately determine initiation and termination sizes in individual cells under those conditions. However, we would not expect to observe any correlations with our analysis method for conditions where a new round of replication initiation is triggered well before the previous one has finished.

### Deletion of *DARS* Can Be Compensated for by Deleting *datA* and Overexpressing *dnaA*.

In addition to RIDA, we have characterized the importance of other pathways involved in the DnaA-ATP/ADP conversion. Specifically, *datA*-dependent DnaA-ATP hydrolysis (DDAH) has been described as being important for DnaA-ATP to ADP conversion (21) and *DARS1, DARS2,* and acidic phospholipids are all suggested to be involved in the conversion of DnaA-ADP into DnaA-ATP (25, 26, 27, 70, 71).

**Fig. 6.**
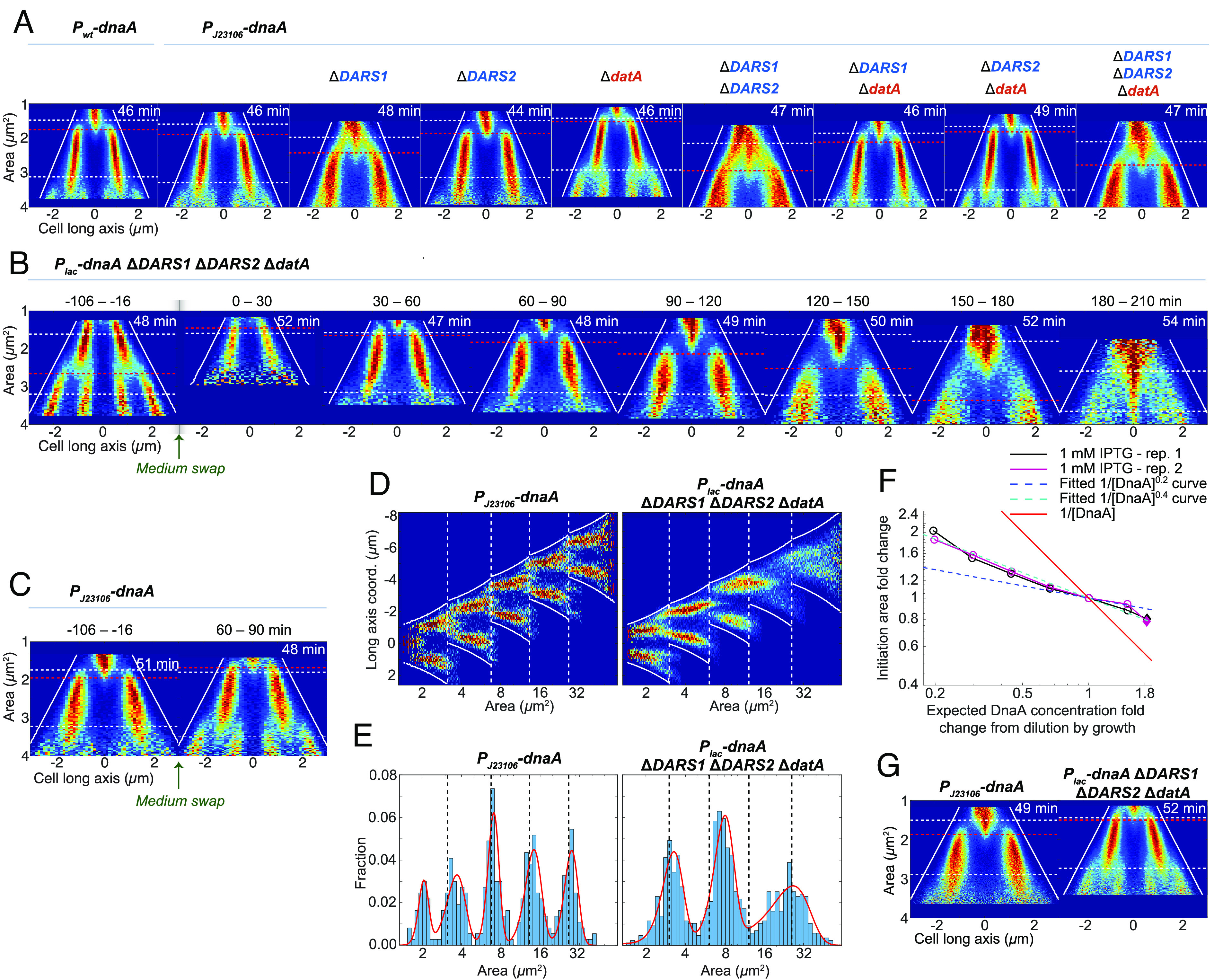
Replication phenotypes of DnaA-ATP/ADP regulatory mutants. (*A*) Fork plots of DnaA-ATP/ADP regulation mutants. The mutations were constructed in the *P*_*J23106*_*-dnaA* background. For similar constructions in the *P*_*wt*_*-dnaA* background, see *SI Appendix*, Fig. S8*A*. Blue text indicates an ADP- to ATP-form converter and red text indicates an ATP- to ADP-form converter. (*B*–*F*) Similar to Fig. 4 but for a Δ*DARS1* Δ*DARS2* Δ*datA* mutant with IPTG-controllable expression of dnaA (*P*_*lac*_*-dnaA*). In *F*, the blue dashed line is the line of the same color in Fig. 4*F* (the strain carrying *P*_*lac*_*-dnaA*), while the turquoise line corresponds to $\frac{1}{{\left[DnaA\right]}^{a}}$ where the exponent a = 0.4. (*G*) Expression of dnaA using 100 µM IPTG in a Δ*DARS1* Δ*DARS2* Δ*datA* mutant compared to the *P*_*J23106*_*-dnaA* strain that constitutively expresses dnaA. Statistics for *A*–*E* and *G* can be found in Dataset S1.

To remove the complexity introduced by the autoregulation of *dnaA* expression, we made deletions of the *DARS1*, *DARS2*, and *datA* loci, as well as combinations of double and triple knockouts, in the *P_J23106_-dnaA* strain. As expected (72, 73), the size at which the cells initiated replication was reduced when *datA* was deleted and increased when *DARS1* or *DARS2* was deleted (Fig. 6*A* and *SI Appendix*, Fig. S8). The *DARS1* deletion mutant gave rise to a particularly broad distribution of initiation sizes. However, the widening of the initiation size distribution did not cause a reduction in the population growth rate for cells grown in bulk (*SI Appendix*, Fig. S8*E*), which would happen if, for example, large variability in initiation size increased the risk of dividing without finishing replication. That the deletion of *DARS1* gave a stronger phenotypic response as compared to *DARS2* is inconsistent with what has been reported previously (26, 32). The inconsistency was not coupled to the autoregulation of *dnaA* as both the width of the initiation size distribution and the average initiation size were larger in the Δ*DARS1* as compared to the Δ*DARS2* strain also in the *P_wt_-dnaA* background (*SI Appendix*, Fig. S8 *A*–*D*). A possible explanation could be a difference in Fis activity since *DARS2* is dependent on Fis to function (31, 74). Similar to Frimodt-Møller et al. (75), we found that deletion of *datA* (Fig. 6*A* and *SI Appendix*, Fig. S8*A*) could partially revert the *DARS* phenotypes. The recent preprint by Boesen et al. suggests that removing *hda* in addition to *datA* can revert the *DARS* phenotypes even further (42). This suggests that the *DARS1* deletion mutant simply has too little DnaA-ATP and implies that the *DARS* and *datA* sites are not critical for replication initiation control as such. The scenario in the *DARS1* deletion mutant is also clearly different from the case where the DnaA concentration was too low (Fig. 4*A*); the latter gives rise to filamentous cells and growth arrest, whereas the former can grow for long periods of time in the microfluidic chip without accumulating filamentous or nongrowing cells.

Regarding other modulators of initiation, we found that a *diaA* deletion gave the same phenotypic response in a *DARS2*:*datA* double-deletion mutant as in the strain having both *DARS2* and *datA* loci present. In both cases, the average initiation size, average division size, and initiation size variability were increased in the Δ*diaA* mutants suggesting that DiaA is not involved in DnaA-ATP/ADP cycling (*SI Appendix*, Fig. S8 *A*–*C*). That Δ*diaA* caused increased initiation and division sizes is in line with previous observations using knockdowns (60). In the study by Camsund et al. (60), the effect of knocking down Fis and IHF was also tested. They found that especially the IHF knockdowns (*ihfA* and *ihfB*) gave strong phenotypic responses, as indicated by a blurring of the branches in the fork plots. This effect was larger than what we observed in the *DARS2:datA* double-deletion mutant presented here, suggesting that the phenotypic effect of knocking down IHF is not due to *DARS2*/*datA* inactivation. For example, IHF also has a role in the assembly of the replication complex at *oriC* (31). The mild effects on initiation from knocking down Fis in the study by Camsund et al. (60) are potentially due to the medium used (succinate + AA) given previous reports of media-dependent phenotypes in Fis knockouts (74). Since Fis binding to *DARS2* is required for *DARS2* function (32, 33), the mild effect of Fis knockdown is consistent with the mild effects of *DARS2* deletion (Fig. 6*A*). However, in addition to being required for *DARS2* function, Fis binds to *oriC* (76), affects the nucleoid structure, and its binding to the chromosome can act as a transcriptional regulator (77), complicating the interpretation of the Fis knockdown’s specific effect on *DARS2*.

To test whether the *DARS* sites are required for making DnaA-ATP in the absence of new DnaA synthesis, we turned off the expression of *dnaA* as described above (Fig. 4, 1 mM IPTG induction), but this time in a mutant lacking *DARS1*, *DARS2,* and *datA*. We found that the cells initiated multiple new rounds of replication after synthesis of DnaA was stopped, also in the absence of *DARS* and *datA* sites (Fig. 6 *B*, *D*, and *E*). Furthermore, the *P_lac_-dnaA* triple mutant was more sensitive than the *P_lac_-dnaA* strain to the concentration of DnaA (Fig. 6*F*), i.e., DnaA-ATP/ADP cycling by *DARS* and *datA* contributes to making initiation robust to variation in DnaA levels. We also found that IPTG-induced (100 µM IPTG) expression of *dnaA* can revert the *DARS* deletion phenotypes (Fig. 6*G*), similarly to what was observed for the deletion of *datA* (Fig. 6*A*). We conclude that the conversion of DnaA-ADP to DnaA-ATP by the *DARS* sites is not the only mechanism responsible for increasing the initiation potential prior to initiation. According to earlier studies (27, 78), a mechanism based on acidic phospholipid–mediated conversion of DnaA-ADP to DnaA-ATP may serve this role. However, when we repressed the expression of *pgsA*, which encodes phosphatidyl glycerophosphate synthase, a critical enzyme needed for making acidic phospholipids (79), cells could still initiate replication without problems, even in a *DARS* deletion background (*SI Appendix*, *Text* and Fig. S9*A*). However, *pgsA* repression caused slight growth defects in both backgrounds (*SI Appendix*, Fig. S9 *B* and *C*). We therefore conclude that the role of acidic phospholipids in replication initiation control is ambiguous.

## Discussion

The results of our experiments shed light on the feasibility of the models described in Box 1. First, we can rule out mechanistic models based on triggering initiation just after cell division (Box 1*D*), since there are growth conditions where initiation typically occurs just before division (Fig. 2).

Considering the inhibitor dilution model (Box 1*A*), the only candidate inhibitor we can think of would be DnaA-ADP, which may compete with DnaA-ATP for binding critical sites at *oriC*. DnaA-ADP is a plausible candidate since it can be made in a stoichiometric relation to the number of chromosomes, for example, if DnaA-ATP is expelled from the DnaA boxes and converted to the ADP-form by RIDA. Making a fixed number of molecules per round of replication creates accuracy in an inhibitor dilution model, where the fixed number of molecules is diluted to the trigger concentration at a well-defined size. However, in vitro experiments have shown that DnaA-ADP promotes rather than inhibits initiation when present together with DnaA-ATP (64, 65, 66).

The initiator accumulation model (Box 1*B*) is not corroborated by Flåtten et al. (16), who show that increasing total DnaA concentration over a range of concentrations does not result in a corresponding change in the amount of chromosomal DNA. Similar experimental data were also reported recently on bioRxiv by Boesen et al. (42). We have failed to confidently quantify the ratios of DnaA concentrations at different levels of expression, but we have found that individual lineages of cells can continue to initiate for several consecutive generations after DnaA synthesis has been turned off. This shows that new synthesis of DnaA is not needed to trigger initiation, which rules out a pure gene expression–based initiator accumulation model. We did not find evidence that autorepression of *dnaA* has a direct role in replication initiation control for the stable growth conditions used here, but it may be indirectly needed in initiation in response to starvation. (p)ppGpp has been suggested to inhibit replication initiation by turning off transcription of *dnaA* (80). Here, autorepression can avoid excessive expression of DnaA such that replication initiation can be kept responsive to (p)ppGpp inhibition of *dnaA* expression.

Our results give the strongest support for an initiator activation/deactivation model (Box 1*C*). In line with (81), we found that regulation of replication initiation is primarily mediated by DnaA-ATP or possibly the DnaA-ATP to DnaA-ADP ratio. First, the cell-to-cell variation in initiation size changes when we disturb mechanisms related to the conversion, i.e., RIDA and *DARS1*. Furthermore, the *DARS* and *datA* mutations can compensate for each other, clearly suggesting that it is the state of DnaA-ATP/ADP binding that is important for initiation and not the absolute concentration of DnaA.

Initiations are repeatedly triggered at a relatively constant size-to-origins ratio in successive generations, even at growth rates with multiple, sometimes overlapping replication cycles. A robust implementation to achieve this type of regulation by the ATP/ADP state of DnaA is to use a modification–demodification cycle (57) where the rate of converting DnaA into its ATP-form is proportional to the cell size and the rate of conversion into the ADP-form is proportional to the number of chromosomes. Similar feedback is implemented by using the aminoacylation state of tRNA for feedback on the expression of amino acid biosynthetic and rRNA operons by measuring the flux balance between amino acid synthesis and consumption in protein synthesis capacity (82). Hda can possibly convert DnaA into its ADP-form with a rate proportional to the chromosome copy number since its activity is dependent on active replication forks (22), the number of which, at least at fast growth, is proportional to the number of chromosomes. We found that the *hda* deletion mutant has clear problems controlling initiation, especially in media that support rapid growth for wild-type cells, where the *hda* deletion mutant eventually stops growing.

When it comes to conversion to the ATP-form, this could be solved by a simple first-order dissociation of ADP and exchange by ATP. This implies a larger increase in the number of DnaA-ATP molecules in larger volumes. If initiation is triggered at a fixed DnaA-ATP concentration, this mechanism would, however, become sensitive to the total concentration of DnaA. Alternative solutions would be that initiation is responsive to the DnaA-ATP/ADP ratio, or the existence of a saturable catalyst mediating the ADP-to-ATP conversion with a total activity proportional to the cell volume. Although repressing *pgsA* did not show any major effect on replication initiation in our hands, the acidic phospholipids may still have this role given their ability to regenerate DnaA-ATP in vitro (27, 78). Both of these mechanisms require additional investigation.

Another caveat with the DnaA-ATP/ADP conversion system is that it cannot be too efficient or accurate, or we would see a sizer phenotype and not the observed correlation coefficient of ~0.5 between consecutive initiations. The observed correlation implies that there is memory in the system, which could, for example, be due to fluctuations in the enzymatic systems performing the conversion on the time scale of the cell cycle.

Finally, we note that RIDA cannot be the main regulator under slow growth conditions where replication is triggered a long time after termination. Most likely, another regulatory scheme is dominating in this regime (47). This mechanism could depend on DnaA-ATP accumulation to a threshold (42), for example through gene expression and titration against DnaA boxes or the *datA* loci (55). To speculate further, it may be that an initiator accumulation model based on DnaA expression and binding to DnaA boxes was the primordial control system for slow-growing ancestors of *E. coli* and that DnaA-ATP/ADP cycling based on RIDA is an adaptation allowing for overlapping replication cycles at fast growth rates.

## Materials and Methods

### Strains and Media.

The strain *E. coli* MG1655 BW25993 *phi80*- *rph+* (EL544) and derivatives thereof were used in all experiments. The genotypes of all strains are listed in *SI Appendix*, Table S5. If not stated otherwise, we have performed our experiments in M9 minimal medium supplemented with 0.06× Pluronic F-108 (Sigma-Aldrich 542342) 0.4% succinate and 1× RPMI 1,640 amino acid solution (Sigma) at 30 °C. All growth media are referred to only by the name of the carbon source and if added amino acids, abbreviated as AA; precise media compositions can be found in *SI Appendix*, Table S1.

#### Chromosomal modifications.

For the construction of chromosomal mutations (deletions, fusions, and replacements), λ Red recombineering (83, 84) was used. The λ Red genes were expressed from plasmid pSIM5-Tet (85). As selectable/counterselectable markers, we used *Acatsac1* (GenBank acc. # MF124798.1) or *Akansac1*, carrying a *cat* gene (conferring chloramphenicol resistance) or a *kan* gene (conferring kanamycin resistance) for positive selection, and the *B. subtilis sacB* gene (conferring sensitivity to sucrose) for negative selection. Mutations were transferred between strains using generalized transduction with phage P1 *vir* (86). For many experiments, the chromosomal construct *cobA*::*seqA-venus* was introduced into the strains, keeping the native *seqA* gene intact. In contrast to having *seqA-venus* as the only source of SeqA in the strains, this mutant was not affected in its exponential growth. Marker- and scar-free knockouts were made using single-strand λ Red or the DIRex method (87). Gibson assembly (88) was used for plasmid construction. See *SI Appendix* for more details.

### Microscopy.

#### Optical configurations.

Five different optical configurations were used. All setups rely on Ti-E (Nikon) microscopes set up for phase-contrast and widefield epi-fluorescence microscopy using 100× oil objectives. Hardware details for each setup are given in *SI Appendix*. Dataset S1 lists which experiments were performed with which microscope configuration.

#### Cell preparation.

For all experiments except when acetate medium was used: The day before each experiment, strains were inoculated into tubes containing the intended media for the experiment from frozen stock cultures stored at −80 °C. Cells were grown overnight in a 30 or 37 °C shaking incubator (200 rpm) depending on which temperature the experiment was performed at. On the day of the experiment, the cells were diluted either 1:100, 1:250, or 1:1,000 in fresh medium and grown for 2 to 4 h before being loaded onto the microfluidic chip, or the cells were loaded from stationary phase into the microfluidic chip. The cells were allowed to grow for 6 to 9 h before starting the experiment if loaded directly from stationary phase or for 2 to 9 h if the cultures were diluted.

For the experiments performed in acetate, the cells were first inoculated from −80 °C into either LB or succinate + AA medium. In LB, the cells were allowed to grow overnight while for succinate + AA medium, the cells were grown for 7 to 8 h, both at 37 °C in a 200 rpm shaking incubator. In both cases, the cells were diluted 1:100 into fresh acetate medium and then grown for 18 to 24 h before being loaded into the chip. The cells were then allowed to grow for roughly 24 h before image acquisition started.

The medium was supplemented with the starting concentrations of IPTG for the experiments using the *P_lac_-dnaA* and *P_lac_-dnaA* Δ*DARS1* Δ*DARS2* Δ*datA* strains, except for the 65 µM experiments, where ON cultures were supplemented with 200 µM IPTG and in the morning of the experiment diluted into medium with 65 µM IPTG.

#### Microfluidic experiments.

Experiments were performed using a PDMS mother machine–type chip with open-ended channels that allowed for the loading of two separate strains (58) in a similar manner as has been described earlier (60). Details of the microfluidic setup and settings for the image acquisitions are given in *SI Appendix*.

### Image Analysis.

#### Image analysis pipeline.

A fully automated image analysis pipeline previously described (60, 89), primarily written in MATLAB (Mathworks), was used. Cell segmentation was performed either with Per Object Ellipse fit (90) or nested Unet neural networks (91, 92). Cell tracking was performed using the Baxter algorithm (93). The wavelet algorithm was used to detect fluorescent foci (94), except for the origin localization experiment (*SI Appendix*, Fig. S1*C*), where radial symmetry was used (95).

#### Postprocessing.

Custom-written MATLAB (Mathworks) functions and scripts were used for processing the pipeline output. For estimation of single-cell initiation and termination sizes, detected foci of either SeqA-Venus or YPet-DnaN were linked together and tracked using the u-track algorithm (61). The parameters were optimized to be able to identify the start of tracks by allowing them to merge and split. To avoid truncation issues when tracking dots, cell lineages consisting of either three, four, or five generations were created. Furthermore, these generations were concatenated into a “supercell” where the area $A\left(t\right)$ at a given time point t is given by $A\left(t\right)=A\left(last\right)+2^{i}\left(a^{c}\left(t\right)-d_{\mathrm{frac}}a^{p}\left(last\right)\right)$ , where A(last) is the area of the supercell before division, i is an index for the generation in the lineage, $a^{c}$ is the area of the cell in generation i , $d_{\mathrm{frac}}$ is the area fraction of the two sisters right after division, and $a^{p}\left(last\right)$ is the area right before division in generation i-1 . To create a supercell, each cell, the cell’s parent, daughters, and sister have to be tracked for a certain number of phase-contrast frames (Dataset S1). Initiation events were defined as the start of a track at least 9 (acetate) or 11 (all other conditions) frames long. Additionally, initiations were not allowed to start on the first 2 frames in a supercell and the 2 adjacent frames to a possible event had to have good segmentation (no gap in cell tracking, a sufficiently high Jaccard index, and no convergence between cells). A further set of criteria were used to define termination events. Here, tracks could not end on the last 2 frames of a supercell. Additionally, tracks had to be located within the boundary of the daughter or in cases where initiation occurred close to division, the long-axis coordinate of the last frame of a trajectory could maximally move 5 pixels from midcell of the current generation. Only supercells that had at least one initiation and termination event and contained unique individual cells except the parent generation were used.

Average initiation sizes and CV were determined by using MATLAB’s fit(‘gauss’) function where the number of Gaussians corresponded to the number of distinct peaks in the total distribution of initiation sizes. To avoid truncation errors, peaks far away from the edges of supercells were chosen. In the *pgsA* repression experiments (*SI Appendix*, Fig. S9*A*) and for DnaQ (Fig. 1*C*), the average initiation size in bulk was determined the same way as in ref. (60).

To get initiation–initiation, initiation–termination, and termination–initiation correlations, all of the events in each supercell were matched with one another, resulting in data points that clustered. Then, clusters corresponding to each type of correlation were chosen manually (*SI Appendix*, Fig. S2). Each cluster was fitted to the sum of a bivariate Gaussian and a constant background. To avoid truncation bias, a cluster that did not correspond to the edges of the supercells was chosen. Furthermore, cells in the supercell structures were all classified as being well segmented (no gaps in cell tracking, a high enough Jaccard index, and that two cell tracks did not converge). The birth–initiation, initiation–division, birth–termination, and termination–division correlations were matched the same way as above.

Initiation size fold change in the DnaA dilution experiments had 1 defined as the bin where the relative difference in initiation size between the IPTG-driven DnaA strain and the reference *P_J23106_-dnaA* strain was the lowest. However, for the experiment labeled “75 µM - rep. 2” in Fig. 4*E*, the third bin was chosen by hand to better match the appearance of the fork plot. The time in Fig. 4*E* was set to 0 at the same bin.

To get the expected DnaA concentration fold change from dilution by growth, the generation times from all bins in the experiment were averaged. The number of normalized generations was then calculated using the average generation time and the normalized times described in above. To calculate the expected concentration, we assumed the concentration of DnaA halves each generation.

*SI Appendix* details on how growth and division correlations, multigenerational fork plots, binned fork plots, and replisome-*ter* distances were acquired.

### Bulk and Rif-Runout Experiments.

Experiments performed on cells in bulk (OD measurements for growth rate determination, NGS and qPCR for *ori*/*ter* ratio determination, RT-qPCR for *dnaA* mRNA abundance determination) and rif-runout experiments are described in detail in *SI Appendix*.

## Supplementary Material

Appendix 01 (PDF)

Dataset S01 (XLSX)

Dataset S02 (XLSX)

Movie S1. Time-lapse of SeqA-Venus fluorescence and phase-contrast images before and after expression of DnaA was turned off. The frame rate was set to 15 frames per second.

## Data Availability

Data and all code have been deposited in the SciLifeLab data repository (96). Proteomics data have been deposited in PRIDE (97). Strains used in this study are available upon reasonable request.
