# Supplementary material for: Regulatory elements coordinating initiation of chromosome replication to the Escherichia coli cell cycle
Source: Proc Natl Acad Sci U S A. 2023 May 23;120(22):e2213795120. doi: 10.1073/pnas.2213795120 (PMC10235992; doi:10.1073/pnas.2213795120)
Supplement: Supplementary file 1 — Appendix 01 (PDF) [file pnas.2213795120.sapp.pdf]

PNAS

Supporting Information for
Regulatory elements coordinating initiation of chromosome replication to the Escherichia coli cell cycle

Anna Knöppel, Oscar Broström, Konrad Gras, Johan Elf & David Fange

Johan Elf & David Fange
Email: johan.elf@icm.uu.se & david.fange@icm.uu.se

This PDF file includes:

- Supporting text
- Figures S1 to S9
- Tables S1–S5
- Legend for Movies S1
- SI References

Other supporting materials for this manuscript include the following:

- Datasets S1 and S2
- Movie S1

Supporting Information Text

Repression of *pgsA* expression does not change the initiation size

We investigated if the source of the DnaA-ATP molecules required to initiate replication is dependent on the acidic phospholipids cardiolipin (CL) and phosphatidylglycerol (PG). *In vitro* experiments have shown that especially CL can promote the dissociation of ADP from the DnaA-ADP complex (1, 2). The *pgsA* gene encodes phosphatidyl glycerophosphate synthase, a protein that catalyzes the biosynthesis of CL and PG (3). Temporarily turning off *pgsA,* gene expression has been shown to stop growth until *pgsA* expression is turned back on (4, 5). Importantly, the effect is not seen in mutants that are not dependent on DnaA for initiation.

We have previously shown that dCas9-mediated repression of *pgsA* expression causes cells to grow ~30% slower while maintaining the same initiation sizes compared to non-repressed cells (6). The impact on growth rate is admittedly lower than for the *pgsA* deletion mutants (4, 5), but if acidic phospholipids are a major contributor to regenerating the ATP-form of DnaA, we would still expect *pgsA* repression to cause increased initiation sizes. Also, since the *DARS* sites are (supposedly) performing the same function as the acidic phospholipids, we would expect *DARS*-deletion mutants to be more sensitive to changes in the acidic phospholipid concentration. However, we found that repressing *pgsA* did not cause any significant change in initiation size in either a reference strain with constitutively expressed *dnaA* or in the *DARS1*:*DARS2* deletion mutant (Figure S9A). The repression of *pgsA* expression also resulted in a similar growth rate decrease in both strains (Figure S9). The *pgsA* mutation may have a slightly larger cell-to-cell variability in the initiation size based on the fork plot, but quantification for the *DARS*-deletion mutant was very difficult. To make sure that we were not selecting for viable cells in the analysis of the microfluidics experiments, we also compared the growth rates in flask cultures (Figures S9B and S9C). Also in the flask experiments, we found that the reference and the *DARS1*:*DARS2* deletion mutant had the same growth rate reduction as a result of *pgsA* repression. Since no major replication phenotype can be seen with repression of *pgsA* expression, although this should have led to a lower concentration of acidic phospholipids and therefore lower DnaA-ATP reactivation as predicted by *in vitro* experiments (1, 2), the role of acidic phospholipids in replication initiation control remains ambiguous.

SI Materials and Methods

Strain construction

The chromosomal knockouts of replication regulators (*DARS1*, *DARS2*, and *datA*) are all marker free and scar free and were made using two different methods: (i) through single strand λ Red, where we cured an inserted selectable/counterselectable marker by an oligo covering the junction of the desired deletion, or (ii), by employing the DIRex method (7). Mutations were transferred between strains through generalized transduction with phage P1. *hda* was deleted through λ Red recombineering of a *kanR* cassette that exchanged the *hda* reading frame. Succinate + AA medium was used for

recovery and selection. All of the other mutant strains were constructed in LB medium. The fact that our *hda* deletion mutant carries an extra *seqA* gene could explain why our strain could be constructed and that it grows well in aerobic conditions (8) whereas the *hda* deletion mutant that carries only one copy of *seqA* had to be constructed in anaerobic conditions to survive (8, 9). Overexpression of SeqA is known to suppress the effects of deleting *hda* (10).

Constitutively expressed DnaA mutant. Firstly, we exchanged an *Acatsac1* cassette in the selectively neutral *galK* locus with a PCR-fragment containing P_{J23106}-*dnaA*, selecting sucrose resistant recombinants. Next, the native *dnaA* gene was deleted by replacing it with *Acatsac1*; thereafter *Acatsac1* was in turn replaced by an oligo containing the J23106 promoter (to allow constitutive expression of the rest of the operon). 123 nt were kept at the end of *dnaA*.

IPTG-inducible DnaA. Two overlapping DNA fragments were simultaneously transformed through electroporation into a strain that expressed the λ Red genes. One fragment was synthesized and contained bp 150–390 of the *lacZ* gene followed by stop codons in all frames, and the *dnaA* gene (including its ribosome binding site) was transcriptionally fused to *mCerulean* MP (no data is shown for mCerulean). The other was a PCR-fragment containing 40 bp homology to the first fragment, a *kan* gene flanked on both sides by Flp recombinase target (FRT) sites, and 40 bp homology to the sequence downstream of the *lacA* gene. The resulting recombinants thus had everything between bp 390 in *lacZ* until just after the *lacA* gene replaced by *dnaA-mcerulean-FRT-kan-FRT*, leaving *dnaA* transcriptionally fused to the beginning of the *lacZ* gene. This construct was transduced to our *seqA-venus* strain and, thereafter, *kan* was removed by expression of Flp recombinase from plasmid pCP20 (11), leaving a scar containing a single FRT site. Finally, the native *dnaA* gene was replaced by the J23106 promoter as described above. The resultant strain is hence dependent on IPTG induction of P_{lac}-*dnaA* for growth. Similarly to the constitutively expressed DnaA mutant, 123 nt were kept at the end of *dnaA*.

CRISPRi/*dCas9.* The system was adapted from (6). *pguide7* plasmids were constructed through Gibson Assembly (12), with the following guide RNAs: *pgsA* (GGAACAGTGTAAGCAACGTA) and *lacO* (control) (TCCGCTCACAATTCCACATG).

oriC labeled with malO-oriC/MalI-Venus. To label *oriC*, λ Red was performed with a PCR-fragment containing an array of 12 *malO* operators and homology to the *yifN* locus, 34.4 kb from *oriC*. *ΔintC::P59-malI-mVenusNB-frt ΔgtrA::SpR* was introduced through P1 transduction. The native *malI* gene was replaced with a *tetR* cassette, also using P1 transduction.

parS/parB terminus tag. This system was adapted from (13) and was recently modified by (14). Here, we transformed *parS* into a neutral position in the terminus region by exchanging y*dbL* for *parS*-FRT-*cat*-FRT. *mCherry-ParBMt1* (Δ*gtrA*::*p58-mCherry-parB-SpR*) (14) was introduced through P1 transduction.

Other chromosomal constructs originated from other labs and were P1 transduced into our strains (*kanR*-*ypet*-*dnaN* and *dnaQ*-*ypet*-FRT-*kanR*-FRT) (15), or introduced in another chromosomal location through λ Red *seqA*-*venus*-FRT-*cat*-FRT (16).

Microscopy

Optical configurations

Five different optical configurations were used, as described below. Dataset S1 lists which experiments were performed with which microscope configuration.

Configuration 1: An inverted TiE (Nikon) microscope with a CFI Plan Apo lambda 1.45/100x oil objective (Nikon) was used. Phase-contrast images were acquired on a DMK 38UX30 (The Imaging Source) camera. Bright-field and fluorescence images were acquired on an iXon Ultra 888 (Andor) camera. TLED+ (Sutter Instruments) with a white LED was used as a light source for phase-contrast and bright-field images. For fluorescence images, the cells were episcopically illuminated by a 5.3 W/cm^2 light beam from a 514 nm Genesis CX-STM 2000 mW (Coherent) laser. The 514 nm laser light was reflected onto the sample using the dichroic mirror Di02-R514 (Semrock) and the emitted fluorescence passed through the same dichroic and was filtered through a BrightLine FF01-474/27 (Semrock) emission filter.

Configuration 2: Similar to Configuration 1, but a red 630 nm LED (Sutter Instruments) was used for phase-contrast, a 600 nm short-pass filter (Edmund Optics) was placed in the microscope camera port for the iXon Ultra, the laser lights were mirrored on FF468/526/596-Di01 (Semrock), and the emitted fluorescence was filtered through a FF01-484/543/702 BrightLine (Semrock) emission filter.

Configuration 3: Similar to Configuration 1, except a 515 nm 06-MLD (Cobolt) laser was used.

Configuration 4: Similar to Configuration 1, but the lasers used for fluorescence imaging were 515 nm (Fandango 150, Cobolt) and 580 nm (VFL, MBP Communications). The laser power was set to 5 W/cm^2 for both lasers. Fluorescence images were acquired using a Kinetix sCMOS (Teledyne Photometrics) camera. The laser lights were reflected onto the sample using a FF444/521/608-Di01 (Semrock) triple-band dichroic mirror, and the emitted fluorescence passed through the same dichroic mirror. The emitted fluorescence was also passed through a BrightLine FF580-FDi02-T3 (Semrock) dichroic beamsplitter. The split fluorescence was then filtered through BrightLine FF01-505/119-25 (Semrock) and BrightLine FF02-641/75-25 (Semrock) filters and focused on two different parts of the sCMOS camera chip. Phase-contrast images were acquired using a DMK 38UX304 (The Imaging Source) camera. A TLED+ (Sutter Instruments) with a 480 nm LED was used as a light source for the acquisition of phase-contrast images. The transmitted light was passed through a FF444/521/608-Di01 (Semrock) triple-band dichroic mirror and reflected onto the camera using a Di02-R514 (Semrock) dichroic mirror.

Configuration 5: Same as in Wiktor et al. (14). Three different settings for the Spectra Gen 3 (Lumencor) were used. TEAL was used for YPet-DnaN with 6% power, YELLOW was used for ParB-mCherry with 4.5% power.

Microfluidic experiments

A PDMS mother machine type chip with open-ended channels was used in the microfluidic experiments. The chip allowed for the loading of two separate strains (10). To keep the medium flowing, pressure on the different ports was maintained with an OB1 MK3+ microfluidic flow controller (Elveflow). This controller was also used to load cells. The microfluidic chips used had four different trap sizes: 875, 1000, 1125, and 1250 nm. Dataset S1 shows which experiments used which trap size. Unless noted, phase-contrast images were acquired every 30 s (100 ms exposure time) and fluorescence images every 2 min (300 ms exposure time).

The duration of the experiments with different carbon sources (Figures 1, 2, S2 and S5; Figure 1 only shows succinate AA) was either 9 h (RDM), 24 h (acetate) or 12 h (all other carbon sources). For the RDM and acetate experiments, phase-contrast images were acquired every 20 s and every 2 min, respectively. Fluorescence images were acquired every 80 s and every 8 min for the RDM and acetate experiments, respectively. The exposure time of the phase-contrast images for all media was set to 50 ms.

The experiments with constitutively expressed DnaA (Figures 3A and 3B; Table S4) and DnaA-ATP/ADP regulatory mutants (Figures 6A and S8A–C) were run for 12–16 h. For the experiments run on Configurations 1 and 3, bright-field images were acquired every 2 min with an exposure time of 100 ms. For the experiments performed on Configuration 2, a bright-field and a corresponding phase-contrast image were acquired before the experiments were started. Bright-field images were used for landmark-based registration.

The duration of the experiments where DnaA expression was turned off was 5.5–6 h (Figures 4, 6B–G and S3C), but divided into two parts. The first part lasted for 90 min, and the succinate + AA medium was supplemented with either 1 mM, 75 μM or 65 μM IPTG. To change to medium without IPTG, new tubing and connectors were used. This required detaching the used tubing and connectors, which was done while medium was still flowing over the cells. Image acquisition was restarted 16–23 min after the first round of imaging finished, and images were then acquired for 4 h. To follow replication, phase-contrast (80 ms exposure time) and fluorescence images (300 ms exposure time) were acquired every 1 and 2 min, respectively.

The experiment P_{lac}-*dnaA ΔDARS1 ΔDARS2 ΔdatA* with constant DnaA expression (induction with 100 μM IPTG) was run for 12 h (Figure 6G). Imaging was done similarly to the experiments where *dnaA* expression was turned off.

For the *pgsA* repression experiments (Figure S9), the medium was supplemented with 50 μg/ml kanamycin at all times. Cells were loaded into a chip with succinate medium that had been further supplemented with 0, 0.5 or 1 ng/ml anhydrotetracycline (aTc). The experiments were run for 8 h.

The Δ*hda* experiments (Figures 5A, 5B, S4A and S4B) were first performed in succinate + AA for 8 h. The medium was then swapped manually to either 0.5× LB or RDM. The swapping procedure was the same as for the experiments where DnaA expression was turned off. Imaging was performed for 8 h with 0.5× LB and 12 h for RDM.
The experiment using an origin marker (Figure S1C) was done the same way as the experiments with a constitutive promoter and DnaA-ATP/ADP regulatory mutants (Figures 3A, 3B, 6A and S8A–C), except it was run for 8 h.

In the replisome-*ter* distance experiments (Figure S6), imaging was performed for 10 h. Phase-contrast images were acquired every 1 min (50 ms exposure time). Fluorescence images were acquired every 1 min (150 ms exposure time), with each acquisition triggering both 580 nm and 515 nm lasers back-to-back by the camera through function generators (Tektronix), one for each laser.

Image analysis

Image analysis pipeline

For configurations 1, 2, 3 and 4, landmark based registration was performed between the two cameras. On Configuration 5, landmark based registration was performed in the replisome-*terminus* experiments between the two different emission filters. To do this, 500 nm fluorescent beads (TetraSpeck, Thermo Fisher) were imaged in both channels.

Post-processing

The correlations between subsequent divisions and growth rate in subsequent generations were determined by extracting cell lineages consisting of two generations and their division sizes and growth rates were matched. These were then bootstrapped with 10 samples. The values in Table S3 correspond to the averages and standard deviations of the bootstrap results.

Multi-generational fork plots were created based on pooled super-cells where the cell long axis position of SeqA-Venus or YPet-DnaN is displayed. The long axis offsets were calculated as the average offset from each super-cell. Additionally, in the experiments where *dnaA* expression was turned off, only the mother cells were included in the analysis (cells stuck to the constriction in each channel). Each mother cell had to be tracked for at least 5 minutes to be included in a super-cell structure.

Binned fork plots were created by sorting fluorescent foci based on their detection time relative to the start of the experiment. To calculate average generation times for each bin, the size expansion of all cells within each bin was used. However, if a cell was tracked for fewer than three frames in a bin, the data points were moved into the bin closest in time. Birth and division sizes were sorted in the bins where cells were born and divided.

To estimate replisome-*ter* distances, detected foci of YPet-DnaN and ParB-mCherry were tracked separately using the u-track algorithm (18), as described in the main Materials and Methods section. Imaging of 100 nm fluorescent beads (TetraSpeck, Thermo Fisher) was performed in both of the fluorescence channels imaged on two different parts of the camera chip for landmark-based registration. Based on this registration, fluorescent foci from the same cell detected in the two channels could be paired. Distances were estimated only between paired YPet-DnaN and ParB-mCherry foci that were the closest to each other. Additionally, each YPet-DnaN focus was only paired with one other ParB-mCherry focus for distance estimation. The tracking of YPet-DnaN was used to determine initiation and termination events. The termination events detected for each cell were used as a reference point to compare distances estimated over time in different cells (Figure S6).

Determination of *ori/ter* ratio using NGS

Overnight cultures of a P_{wt}-*dnaA* strain without any fluorescent marker on the replisome were grown in 1 ml medium (LB, succinate + AA, and glucose + AA) at 37 °C. The cultures were diluted at least 250-fold in 25 ml fresh media pre-heated to 30 or 37 °C and grown to OD_{600} = 0.2 (LB 30 and 37 °C), 0.17 (succinate + AA 37 °C), and 0.14 (succinate + AA and glucose + AA 30 °C) whereafter rifampicin was added to the final concentration of 0.3 mg/ml and any ongoing transcription was allowed to terminate by leaving the cultures in the shaking incubator for 2 min before withdrawing 2× 1.5 ml samples. The cells in the withdrawn samples were quickly pelleted at room temperature and frozen at -84 °C (*i.e.,* the cells were frozen within 4 min after adding rifampicin). In addition, 0.5 ml of a sample with stationary cells grown in LB at 37 °C for 1.5 days was pelleted and frozen. DNA was prepared using the MasterPure™ Complete DNA and RNA purification kit (Lucigen), according to the manufacturer's instructions. Illumina libraries were prepared using the TruSeq DNA PCR-Free library kit and the samples were sequenced using one lane of a MiSeq run at SciLifeLab Uppsala. TrimGalore (19) was used to remove adapter sequences and Bowtie2 (20) to map the sequencing reads over the reference genome of the P_{wt}-*dnaA* strain mentioned above. The genome position of the first base of each mapped read was binned into either ~30 kb or ~1.5 kb large bins (Figure S7).

Determination of *ori/ter* ratios using qPCR

The DNA samples used in the qPCR experiment presented in Figure S7 were the same as the ones prepared for the NGS Marker Frequency analysis in the same figure. Quantitative PCRs were performed with 0.1 and 1 μl template and in technical duplicates for primer pair *ori_3* and single PCRs for primer pair *ter_14*. The technical replicates were averaged and the *ori*/*ter* ratio was calculated according to $2^{-\Delta CT}$. The primers used in the qPCR were *ori_3_f/r* and *ter_14_f/r* (21) (Dataset S2).

RT-qPCR

Overnight cultures were grown in 1 ml medium supplemented with 1 mM IPTG. The cultures were diluted to 1:100 in 25 ml fresh pre-heated media supplemented with 1 mM IPTG. For the open symbols shown in Figure S3A cells were grown to OD_{600} = 0.2 after which samples for normalization were withdrawn. The cells in the remainder of the cultures were carefully washed four times through centrifugation in media without IPTG. Then the cells were resuspended in 100 ml pre-heated medium and grown for a total of four hours at 30 °C with shaking. Samples were withdrawn at different times. For the closed symbols in Figure S3A cells were grown to OD_{600} = 0.1 after which they were centrifuged to a pellet and resuspended in 50 ml of medium. After resuspension the cell cultures were incubated for the time given in the figure.

RNA was prepared using the PureLink RNA Mini Kit (ThermoFisher) and DNase treated using the Turbo DNA-free kit (Invitrogen). The RNA was reverse-transcribed into cDNA through the High Capacity Reverse Transcription kit (ThermoFisher), and the Power SYBR Green PCR mix (ThermoFisher) was used for the quantitative PCR reactions according to the manufacturer's instructions. Quantitative PCRs were performed in technical replicates on all samples, using primer pairs for *dnaA* and the reference gene

cysG. The fold change was calculated according to the $2^{-\Delta\Delta CT}$ method (22). The primers used in RT-qPCR were *cycG_f/r* (23) and *dnaA_f/r* (Dataset S2).

Rifampicin (rif)-runout experiments

Experiments were carried out essentially as described in (24). More specifically, overnight cultures in succinate + AA medium at 30 °C (made in quadruplicates for the experiment in Figure 3C and duplicates for Figure S8D) were diluted to grow for at least 10 generations in a shaking incubator before reaching $OD_{600} \approx 0.05$. To stop initiation and division, rifampicin and cephalexin were added to the final concentrations of 0.3 mg/ml and 0.01 mg/ml, respectively (from rifampicin stock 30 mg/ml in DMSO and cephalexin stock 10 mg/ml in H_2O). Since any ongoing replication is still active after adding the antibiotics, the replication forks will go on until termination, allowing the chromosome copy number to serve as a measurement of *oriC* number at the time of adding the antibiotics. After 4–5 h of further incubation in the shaking incubator, 5 ml of the samples were fixed in 50 ml ice cold 70% EtOH and stored at -20 °C.

For flow cytometry analysis, cells in 1 ml aliquots of the stored samples were spun down and resuspended in 1 ml of 50 mM Tris-Mg (pH 7.5) buffer supplemented with 10 mM $MgSO_4$. The cells were concentrated through centrifugation, and 0.8 ml of the supernatant was removed. 1–2 μl SYTOX green was added to stain the DNA in the cells, and the DNA content in the fixed cells was analyzed with a MACSquant Analyzer Flow Cytometer.

Growth rate determination in a plate reader

The measurements were performed by diluting overnight cultures grown at 30 °C in succinate + AA medium 1:1000 in fresh medium and, thereafter, the increase in optical density (OD_{600}) over time was measured using a Bioscreen C Reader (Oy Growth Curves) with shaking. Doubling times during the exponential phase were found by regression to $OD(t) = b + c \times 2^{\frac{t}{\tau}}$ where τ is the doubling time.

Determination of growth rates in E-flasks

Deletion of hda

Overnight cultures in duplicates of the Δ*hda* and P_{wt}-*dnaA* strains were grown in succinate + AA medium at 30 °C. The cultures were diluted 1:5000 while OD_{600} was monitored until ≈ 0.02 with a Visible Spectrophotometer, PV4 (VWR). At this point, the medium was swapped to 0.5× LB by diluting 1:50 into pre-warmed 0.5× LB medium. At OD_{600}≈ 0.13 the reference cultures were re-diluted 1:50 in 0.5× LB medium. During analysis, we corrected for dilution factors.

Turning off dnaA expression

Overnight cultures of the P_{lac}-*dnaA* and P_{wt}-*dnaA* strains were grown in succinate + AA medium supplemented with 1 mM IPTG at 30 °C. The next day the cultures were diluted 1:100 into pre-warmed medium and allowed to grow to OD_{546} = 0.1 while growth was monitored. To remove IPTG from the cells, the cultures were first centrifuged at 4000 rcf for 4 minutes and then resuspended in pre-warmed medium without any IPTG, except for one culture where the resuspension was performed with medium supplemented with 1 mM IPTG. OD_{546} was then monitored approximately every 30 minutes for approximately 500 minutes. Cultures were kept approximately within an OD_{546} range of 0.01–0.1. To adjust for dilutions, the target OD_{546} (0.01) was divided by the OD_{546} value last measured before the dilution.

Repression of pgsA

Overnight cultures of EL3242 (P_{J23106}-*dnaA lacO* gRNA), EL3244 (P_{J23106}-*dnaA pgsA* gRNA) EL3298 (P_{J23106}-*dnaA lacO* gRNA Δ*DARS1* Δ*DARS2*), and EL3299 (P_{J23106}-*dnaA pgsA* gRNA Δ*DARS1* Δ*DARS2*) were grown in succinate + AA medium supplemented with 50 *µ*g/ml Kan at 30 °C. Cultures were diluted at least 1:20000 into pre-warmed medium ±1 ng/ml aTc. The cultures were allowed to grow for ~16 h before re-dilution into the same medium (pre-warmed) to a final OD_{600} of about 0.005 and OD_{600} was monitored over time. The experiment was repeated three times.

Proteomic analysis with mass spectrometry

The proteomic analyses were performed as described in (25). Briefly, cultures were started by inoculating a 50 ml succinate medium with 250 *µ*l overnight culture. The cultures were allowed to grow to OD_{600} = 0.2, pelleted by centrifugation, and washed twice in PBS. The pellets were frozen before further preparation. The proteomics core facility at the Sahlgrenska Academy, Gothenburg University, homogenized the samples, digested with trypsin and labeled the peptides with TMT 10-plex isobaric tagging reagents, and performed relative quantification of peptides by LC-MS/MS, as described in (25). The mass spectrometry proteomics data have been deposited to the ProteomeXchange Consortium via the PRIDE (26) partner repository with the dataset identifier PXD036580.

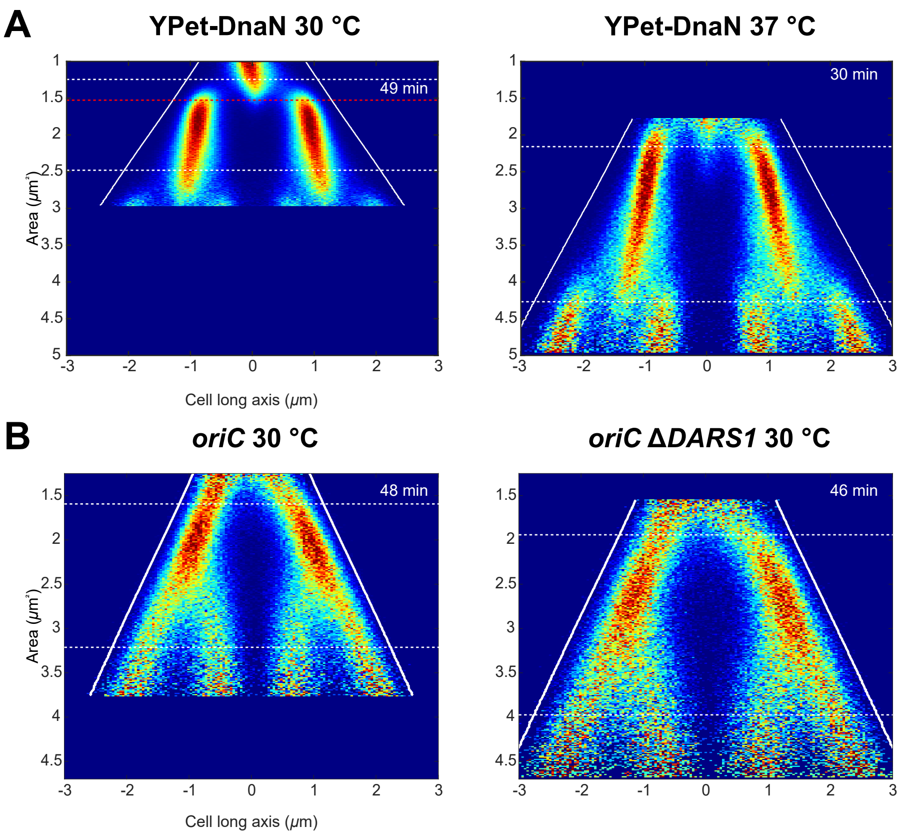

Figure S1. Replication and its temperature dependence and the localization of oriC. (A) *The strain carrying YPet-DnaN and parS-ter/ParB-mCherry was grown at 30 and 37 °C.* ***(B)*** *Strains with oriC labeled using malO-oriC/MalI-Venus. One of the strains also has DARS1 deleted. All cells were grown in succinate + AA medium. For statistics see Dataset S1.*

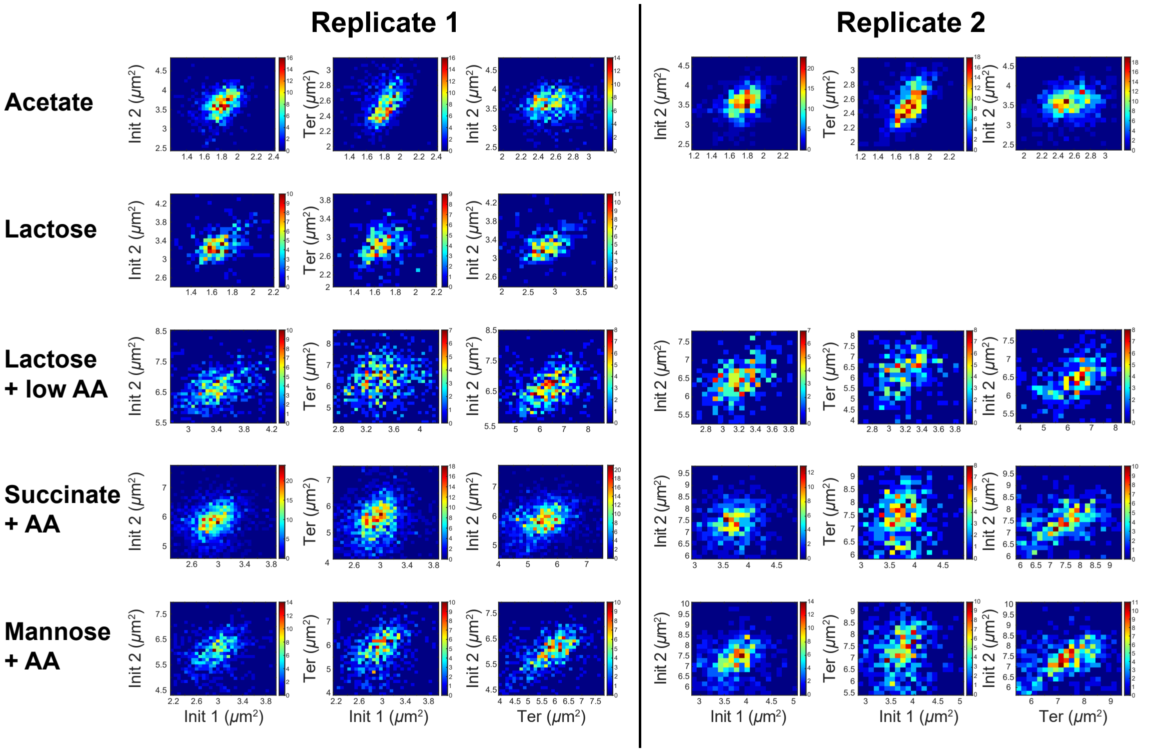

Figure S2. Termination-initiation correlation clusters. *Matched initiation and termination events from super-cells clustered for all growth conditions where correlations between replication events were determined. The colorbar for each plot shows the number of matched events in each bin. The replicates for each medium are separated by the vertical black line. Statistics can be found in Dataset S1.*

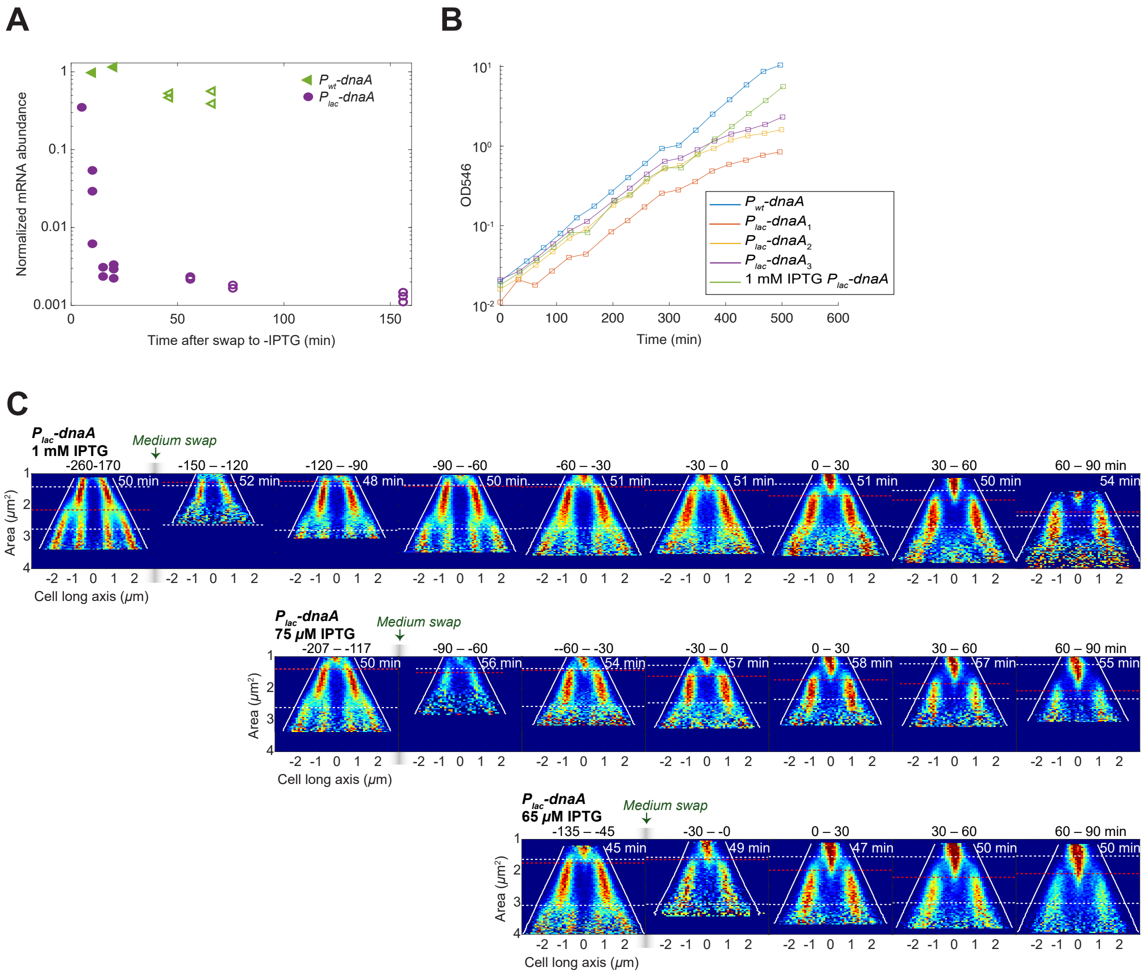

***Figure S3**. **Effects on expression of dnaA, growth and replication initiation after removal of IPTG from the P_{lac}-dnaA strain**. **(A)** RT-qPCR. Relative dnaA mRNA levels at varying time points before and after swapping from medium with 1 mM IPTG to medium lacking IPTG. Cells were kept at 30 °C in succinate + AA medium. For closed symbols the values are related to cells from the same strain resuspended in media with 1 mM IPTG. For open symbols the values are related to cells before medium swap. Closed purple symbols are the results from three replicates of the same experiment (time points of measurement vary between replicates). Closed green symbols are results from a single experiment. Open symbols are the results of multiple clones of the same strain in one experiment. **(B)** OD_{546} measurements from flask cultures of the P_{wt}-dnaA and P_{lac}-dnaA strains after swapping from medium with 1 mM IPTG in succinate + AA to medium without IPTG. Three different replicates of the P_{lac}-dnaA strain without IPTG as well as a control with IPTG were used. **(C)** Fork plots after the removal of IPTG from the media for the P_{lac}-dnaA strain. Top row: starting from 1 mM IPTG (same data as in Figure 4A). Middle row: starting from 75 μM IPTG. Bottom row: starting from 65 μM IPTG. The fork plots have been aligned time-wise so that time 0 is when the initiation phenotype is the most similar to P_{J23106}-dnaA. Statistics for (C) can be found in Dataset S1.*

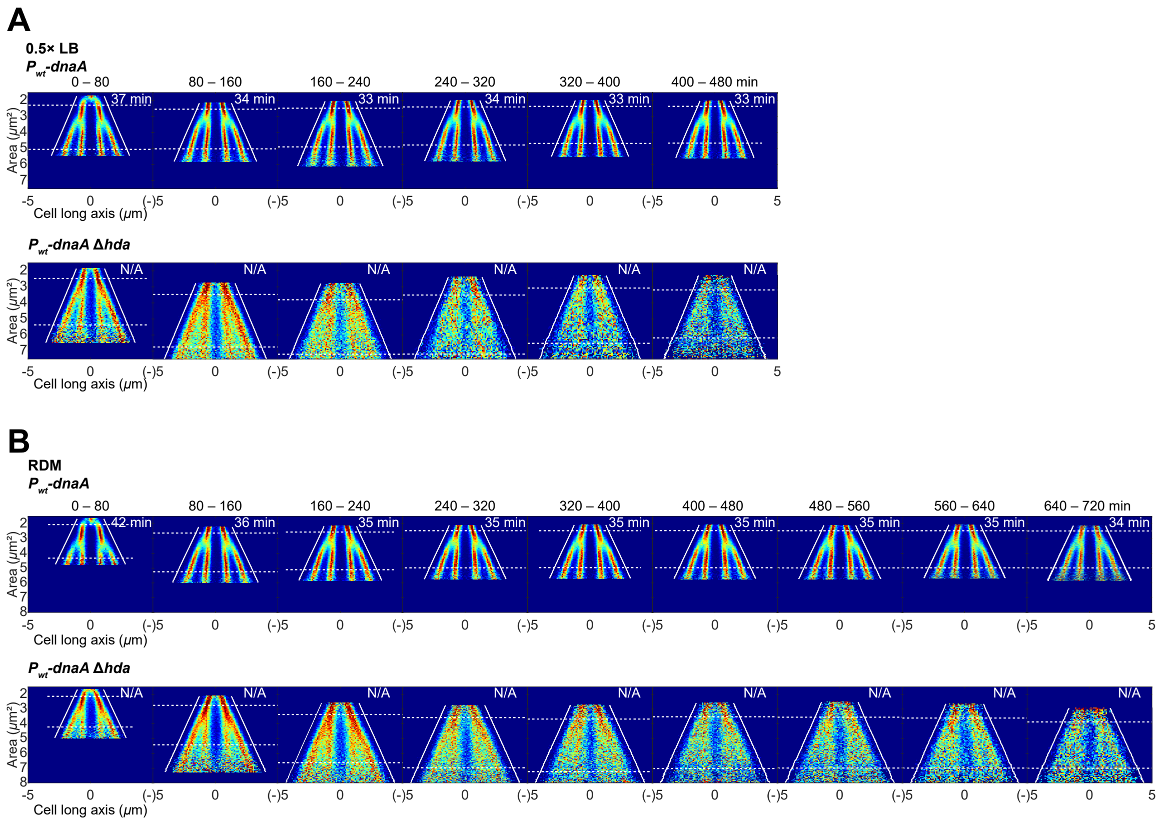

Figure S4. Time-lapse fork plots of a Δhda strain after swapping from succinate + AA to richer media. (A) *Fork plots for the P_{wt}-dnaA and Δhda strains for the full experimental duration after swapping from succinate + AA medium to 0.5× LB medium.* ***(B)*** *Same as (A) but the swap was to RDM. Statistics can be found in Dataset S1.*

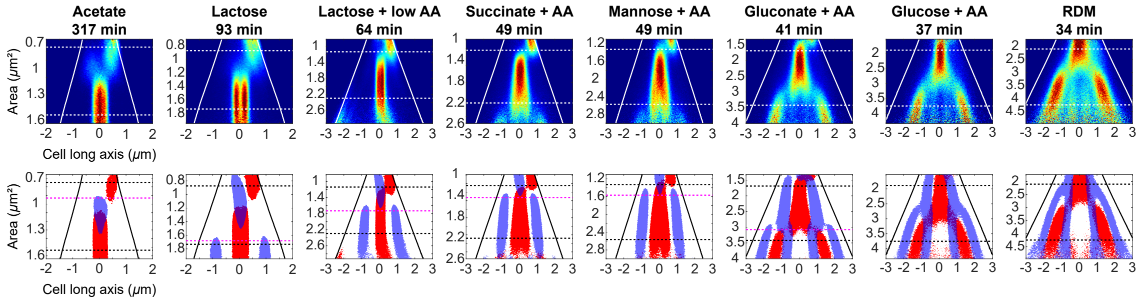

Figure S5. Replisome-terminus fork plots for different carbon sources. *Fork plots of a strain that simultaneously carries fluorescent markers for replication (a YPet-DnaN translational fusion; Figure 2) and terminus (the ParB-mCherry/parS system, where parS is placed in the terminus region; top row). In the bottom row, the results from YPet-DnaN (blue; Figure 2A) have been superimposed onto the results from the terminus marker (red). The results are displayed as filled contours. Lines as in Figure 1B, except white lines are now black and the red line is changed to magenta for visibility. Statistics can be found in Dataset S1.*

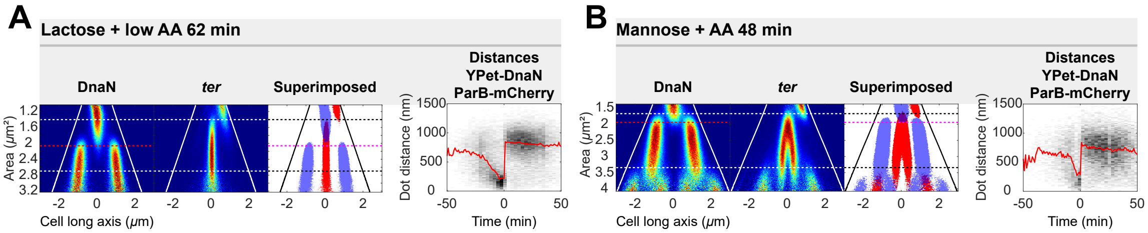

Figure S6. Replisome-terminus distances. *Distances between replisomes and termini in single cells grown in either (A) lactose + low AA or (B) mannose + AA medium.* ***(A & B):*** *From left to right in both panels: (i) Fork plot of a strain that carries fluorescent markers for replication (a YPet-DnaN translational fusion). (ii) Fork plot of a strain that carries fluorescent markers for terminus (the ParB-mCherry/parS system, where parS is placed in the terminus region). (iii) Filled contours of the two leftmost fork plots have been superimposed. The lines and colors are the same as in Figure S5. (iv) Heat-map of 2D histograms for distances between YPet-DnaN and ParB-mCherry foci in single cells and relative time from YPet-DnaN track disappearance. Red lines indicate the median distance over time. Statistics can be found in Dataset S1.*

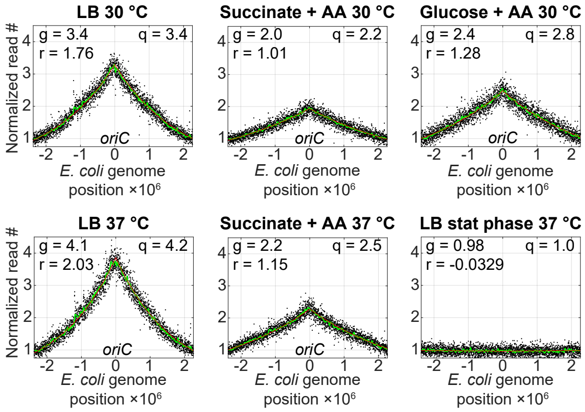

Figure S7*. *Marker frequency analysis. *Normalized number of mapped reads from NGS (y-axis) for each chromosome region (x-axis) for a wild-type strain grown at different conditions. The genome coordinate is centered around oriC. The number of reads was binned into regions of either ~1.5 kb (black dots) or ~30 kb (green line) and normalized to the leftmost bin in each case. The red lines are the results of regressions of the 30 kb binned normalized number of reads (green line), N, to the function $N(x) = b \times 2^{-rx}$ where, x is a normalized genome coordinate in which $x = 0$ at oriC and $x = 1$ on the opposite side of the chromosome. r is interpreted as the ratio between the time spent replicating the chromosome (C-period) and the cell generation time (27). Inset in each panel: oriC/ter (g) ratios are calculated from regression as $g = \frac{N(0)}{N(1)}$. qPCR determinations of oriC/ter ratios (q) are shown to the right in each panel. The reported values are the mean of the values from two oriC qPCR replicates divided by one ter qPCR replicate.*

A

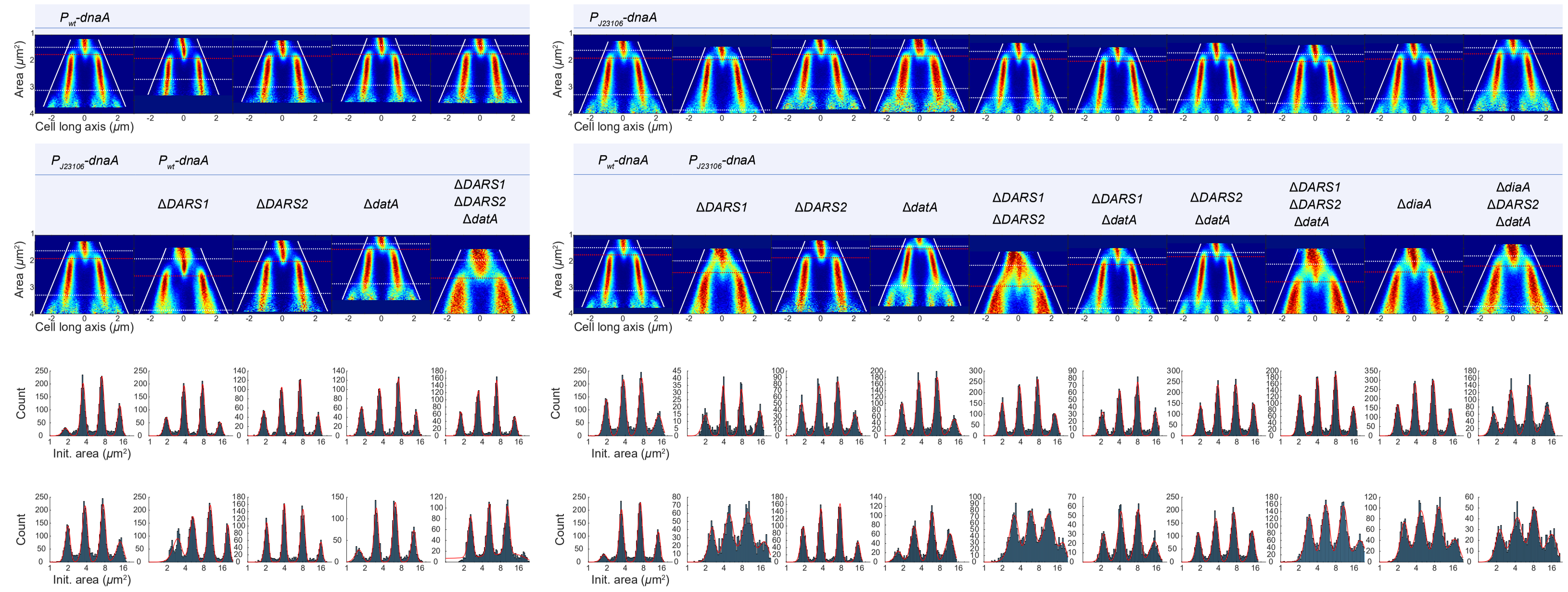

C

	P_{wt}-dnaA Mean	CV	ΔDARS1 Mean	CV	ΔDARS2 Mean	CV	ΔdatA Mean	CV	ΔDARS1 ΔDARS2 ΔdatA Mean	CV
**Init. area ref. (μm²)**	1.75	0.12	1.90	0.11	1.81	0.10	1.74	0.11	1.73	0.11
**Init. area mut. (μm²)**	1.89	0.13	2.55	0.13	2.00	0.09	1.54	0.11	2.63	0.11
**Birth area ref. (μm²)**	1.48	0.18	1.46	0.18	1.46	0.15	1.40	0.18	1.42	0.17
**Birth area mut. (μm²)**	1.59	0.20	1.90	0.21	1.52	0.18	1.33	0.18	1.95	0.19
**Division area ref. (μm²)**	3.12	0.15	2.70	0.16	2.99	0.14	2.92	0.16	2.95	0.16
**Division area mut. (μm²)**	3.28	0.17	3.86	0.16	3.22	0.16	2.85	0.16	4.07	0.18
**Doubling time ref. (min)**	46	0.10	51	0.10	47	0.07	44	0.08	45	0.07
**Doubling time mut. (min)**	46	0.09	46	0.10	45	0.08	44	0.08	44	0.07

	P_{J23106}-dnaA Mean	CV	ΔDARS1 Mean	CV	ΔDARS2 Mean	CV	ΔdatA Mean	CV	ΔDARS1 ΔDARS2 Mean	CV	ΔDARS1 ΔdatA Mean	CV	ΔDARS2 ΔdatA Mean	CV	ΔDARS1 ΔDARS2 ΔdatA Mean	CV	ΔdiaA Mean	CV	ΔdiaA ΔDARS2 ΔdatA Mean	CV
**Init. area ref. (μm²)**	1.89	0.13	1.95	0.10	1.75	0.13	1.81	0.13	1.92	0.12	2.01	0.12	1.96	0.12	2.02	0.12	1.93	0.12	1.73	0.17
**Init. area mut. (μm²)**	1.75	0.12	2.43	0.24	1.86	0.11	1.52	0.18	2.94	0.23	2.12	0.16	1.82	0.15	2.78	0.21	2.40	0.25	2.17	0.28
**Birth area ref. (μm²)**	1.59	0.18	1.84	0.20	1.49	0.20	1.50	0.21	1.63	0.19	1.83	0.18	1.69	0.20	1.75	0.19	1.66	0.18	1.50	0.20
**Birth area mut. (μm²)**	1.48	0.18	1.98	0.21	1.49	0.18	1.42	0.21	2.15	0.20	1.86	0.18	1.66	0.19	2.10	0.21	1.99	0.23	1.79	0.23
**Division area ref. (μm²)**	3.28	0.17	3.87	0.17	3.06	0.17	3.05	0.19	3.40	0.16	3.83	0.15	3.48	0.17	3.61	0.16	3.44	0.16	3.13	0.17
**Division area mut. (μm²)**	3.12	0.15	4.07	0.18	3.14	0.17	2.92	0.19	4.35	0.18	3.79	0.15	3.50	0.17	4.32	0.18	4.07	0.20	3.71	0.20
**Doubling time ref. (min)**	46	0.09	49	0.07	45	0.08	45	0.09	46	0.07	44	0.06	50	0.07	47	0.06	44	0.06	43	0.07
**Doubling time mut. (min)**	46	0.10	48	0.07	44	0.08	46	0.10	47	0.07	46	0.07	49	0.07	47	0.08	45	0.06	43	0.06

D

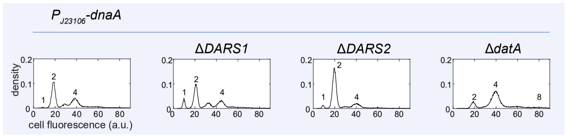

E

P_{J23106}-dnaA	-	ΔDARS1	ΔDARS2	ΔdatA	ΔDARS1 ΔDARS2	ΔDARS1 ΔdatA	ΔDARS2 ΔdatA	ΔDARS1 ΔDARS2 ΔdatA	ΔdiaA	ΔdiaA ΔDARS2 ΔdatA
Doubling time (min)	46.4 ± 0.8	47.7 ± 0.07	47.5 ± 0.49	46.4 ± 0.23	48.7 ± 0.75	46.2 ± 0.21	47.1 ± 0.26	48.9 ± 1.39	49.0 ± 0.33	47.7 ± 0.64

Figure S8. Comparison of different DnaA-ATP/ADP regulatory mutants with wild-type and constitutive expression of DnaA*. (A–C)** *Microfluidic experiments.* ***(A) *Fork plots of different mutants constructed in P_{wt}-dnaA background (left section) and P_{J23106}-dnaA background (right section; same as Figure 6A). As shown in Figure 6A, with the addition of the reference strain that was included in the same experiment, is shown on the top row.* ***(B)*** *Same as Figure 3B, but for strains in (A).* ***(C)*** *Single-cell statistics for the listed strains. Initiation sizes are derived as an average from a single peak in the distributions found in (B). CVs were calculated from the average and standard deviation of the same peaks. Birth sizes, division sizes and generation times come from the cells in the fork plots in (A).* ***(D)*** *Same as Figure 3C, but for the indicated mutation.* ***(E)*** *Average growth rate (± SEM) of regulatory mutants grown in a Bioscreen plate reader. The average and SEM is for three clones for each of the strains. All clones of all strains were grown in the same plate. (A–E): In all experiments, cells were grown in succinate + AA medium at 30 °C. Statistics for panels (A–C) can be found in Dataset S1.*

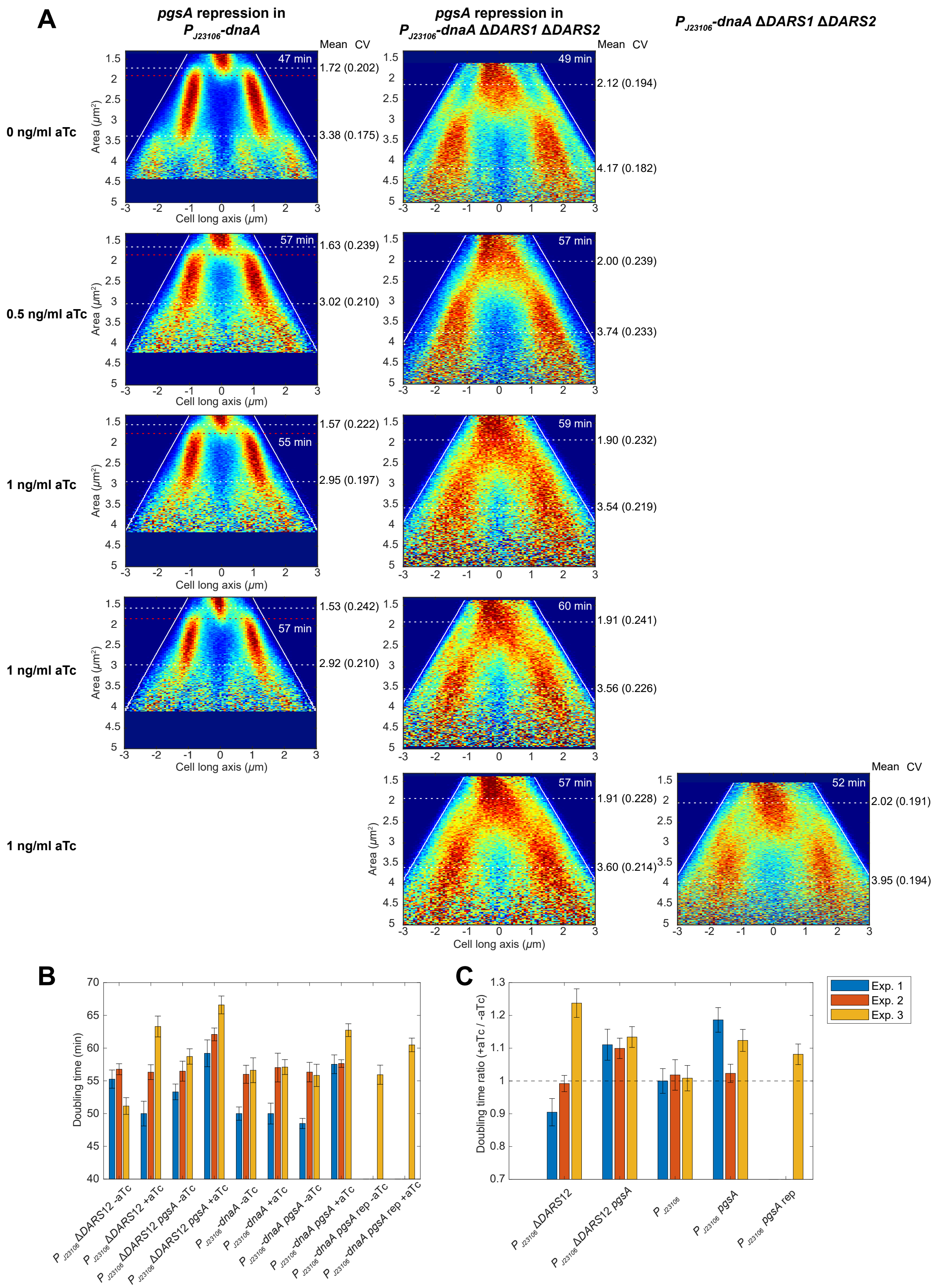

***Figure S9. pgsA repression in the presence and absence of DARS1 and DARS2. (A)** Fork plots showcasing the effects of repressing pgsA expression in a P_{J23106}-dnaA and P_{J23106}-dnaA ΔDARS1 ΔDARS2 at different levels. Additionally, a control without pgsA repression in the ΔDARS1 ΔDARS2 background is included. **(B)** Experiment where the doubling times of the*

strains in (A) were determined using OD measurements of cells grown in E-flasks without (-aTc) and with (+aTc) pgsA repression at 1 ng/ml aTc concentration. This also includes a strain with intact DARS1 and DARS2 loci and without the pgsA repression system (P_{J23106}-dnaA). The experiment was performed in succinate + AA medium at 30 °C. Error bars indicate 67% confidence intervals of the doubling time estimate in each experiment. Different colors of bars indicate three biological replicates. Exp3 also includes technical replicates of pgsA repression in the P_{J23106}-dnaA P_{pGuide}-pgsA strain. ***(C)*** *Ratios of doubling times with (+aTc) and without (-aTc) pgsA repression of the data shown in (B). Error bars indicate 67% confidence intervals for each of the ratios. Statistics for (A) can be found in Dataset S1.*

Table S1. *Media.*

**Medium**	**Supplement**[a]	**Abbreviation**
M9 minimal medium	0.4% succinate and 1× RPMI 1640[b]	Succinate + AA[bc]
M9 minimal medium	0.4% acetate	Acetate
M9 minimal medium	10 mM lactose	Lactose
M9 minimal medium	10 mM lactose and 0.025× RPMI 1640[b]	Lactose + low AA[b]
M9 minimal medium	20 mM mannose and 1× RPMI 1640[b]	Mannose + AA[b]
M9 minimal medium	20 mM gluconate and 1× RPMI 1640[b]	Gluconate + AA[b]
M9 minimal medium	0.4% glucose and 1× RPMI 1640[b]	Glucose + AA[b]
RDM	-	RDM
LB	-	LB
0.5× LB	Sigma H_2O	0.5× LB

[a] We additionally supplemented all media used in microscopy experiments with the surfactant Gibco® Pluronic® F-108. 0.18× (51 μg/ml) for RDM and 0.5× LB, and 0.06× (15.3 μg/ml) for all other media.

[b] AA is used as an abbreviation for RPMI 1640 Amino Acids Solution (50×; R7131 Sigma-Aldrich).

[c] Used in experiments in most figures.

Table S2. *Statistics of single-cell measurements of the cells shown in Figure 2. Initiation sizes could not be determined for cells grown in glucose + AA and RDM.*

Medium	Initiation area (μm^2)	Initiation area CV	Initiation area to origin ratio (μm^2)	Division area (μm^2)	Division area CV
Acetate	0.93	0.11	0.93	1.54	0.08
Acetate[a]	0.91	0.12	0.91	1.49	0.08
Lactose	1.68	0.10	0.84	1.73	0.13
Lactose + low AA	1.73	0.15	0.865	2.29	0.18
Lactose + low AA[a]	1.63	0.10	0.815	2.19	0.16
Succinate + AA	1.53	0.10	0.765	2.48	0.16
Mannose + AA	1.57	0.11	0.785	2.56	0.14
Gluconate + AA	3.08	0.10	0.77	3.41	0.14
Glucose + AA	N/A	N/A	N/A	3.75	0.12
RDM	N/A	N/A	N/A	4.24	0.14

[a] repeat experiment

***Table S3**. Correlations between initiation, termination, division, and growth rate for cells grown in different media. $\rho_{I,I}$ is the correlation coefficient between cell sizes at two subsequent initiations . $\rho_{B,I}$ is the correlation coefficient between cell sizes at birth and the subsequent initiation, $\rho_{I,D}$ for initiation and the subsequent division, $\rho_{B,T}$ birth and the next coming termination and $\rho_{T,D}$ for termination and the subsequent division. $\rho_{D,D}$ is the correlation coefficient between cell sizes at two subsequent divisions. $\rho_{G,G}$ is the correlation coefficient between growth rates in two consecutive generations. For $\rho_{I,I}$, $\rho_{B,I}$, $\rho_{I,D}$, $\rho_{B,T}$ and $\rho_{T,D}$ ± indicates 67% confidence intervals as in Figure 5C. For $\rho_{D,D}$ and $\rho_{G,G}$ ± indicates the standard deviation of the coefficients from 10 bootstrap samples. Statistics can be found in Dataset S1.*

**Medium**	$\rho_{I1,I2}$ [b]	$\rho_{B,I}$ [b]	$\rho_{I,D}$ [b]	$\rho_{B,T}$ [b]	$\rho_{T,D}$ [b]	$\rho_{D,D}$ [c]	$\rho_{G,G}$ [c]
Acetate	0.46 ± 0.02	0.33 ± 0.03	0.62 ± 0.02	0.25 ± 0.03	0.49 ± 0.03	0.40 ± 0.01	0.41 ± 0.01
Lactose	0.38 ± 0.04	0.26 ± 0.05	0.86 ± 0.01	0.34 ± 0.05	0.50 ± 0.04	0.52 ± 0.01	0.19 ± 0.01
Lactose + low AA	0.47 ± 0.04	0.31 ± 0.04	0.45 ± 0.04	0.22 ± 0.04	0.51 ± 0.04	0.41 ± 0.01	0.15 ± 0.004
Succinate + AA	0.40 ± 0.02	-0.02 ± 0.02	-0.04 ± 0.02	-0.07 ± 0.03	0.04 ± 0.03	0.56 ± 0.01	0.35 ± 0.01
Mannose + AA	0.55 ± 0.03	0.07 ± 0.03	0.14 ± 0.03	0.04 ± 0.04	0.11 ± 0.05	0.52 ± 0.01	0.28 ± 0.01
Acetate[a]	0.40 ± 0.03	0.22 ± 0.04	0.57 ± 0.02	0.25 ± 0.03	0.57 ± 0.02	0.32 ± 0.02	0.40 ± 0.01
Lactose + low AA[a]	0.43 ± 0.06	0.11 ± 0.06	0.04 ± 0.05	0.15 ± 0.05	0.48 ± 0.03	0.37 ± 0.01	0.21 ± 0.01
Succinate + AA[a]	0.28 ± 0.05	-0.02 ± 0.04	-0.02 ± 0.04	-0.01 ± 0.06	-0.12 ± 0.06	0.49 ± 0.02	0.38 ± 0.01
Mannose + AA[a]	0.49 ± 0.04	-0.02 ± 0.04	0.01 ± 0.04	-0.08 ± 0.08	0.06 ± 0.06	0.54 ± 0.02	0.22 ± 0.07

[a] repeat experiment

[b] ± indicates 67% confidence intervals

[c] ± indicates SD from bootstrapping

Table S4*. (Top) Bulk measurements of doubling time (OD in plate reader), dnaA mRNA levels (RT-qPCR) and protein levels for DnaA, DnaN and RecF (LC-MS/MS) in strains carrying either constitutive (P_{J23106}-dnaA) or wt (P_{wt}-dnaA) expression of dnaA. Values are the averages of either three clones with two technical replicates per clone (P_{wt}-dnaA, doubling time) two clones with two technical replicates per clone (P_{J23106}-dnaA, doubling time), or two (RT-qPCR and LC-MS/MS) clones for the same strains in one experiment for each type of measurement. ± indicates SEM. (Bottom) Single-cell measurements (microscopy) of averages and CVs for the distributions of initiation sizes, birth sizes, division sizes, and generation times for the P_{wt}-dnaA and P_{J23106}-dnaA strains. The values presented are the averages from 6 independent repeats for the P_{wt}-dnaA strain (from Figure S8A) and 12 independent repeats for the P_{J23106}-dnaA strain (most experiments are included in Figure S8A). ± indicates SEM.*

	P_{wt}-dnaA	***P_{J23106}-dnaA***
Doubling time (min)[a]	46.6 ± 0.56	45.5 ± 0.18
[*dnaA* mRNA][a]	1	1.5
[DnaA protein][a]	1	0.99
[DnaN protein][a]	1	2.76
[RecF protein][a]	1	2.27
Initiation area (μm^2)[b]	1.80 ± 0.02	1.89 ± 0.03
Initiation area CV[b]	0.11 ± 0.003	0.12 ± 0.005
Birth area (μm^2)[b]	1.50 ± 0.03	1.66 ± 0.04
Birth area CV[b]	0.17 ± 0.005	0.19 ± 0.003
Division area (μm^2)[b]	3.07 ± 0.12	3.45 ± 0.08
Division area CV[b]	0.15 ± 0.002	0.17 ± 0.003
Generation time (min)[b]	46 ± 1	46 ± 1
Generation time CV[b]	0.08 ± 0.006	0.07 ± 0.003

[a] bulk measurement

[b] single-cell measurement

Table S5. Strain list.

Strain number	Strain	Genotype	Ref	Whole genome sequenced[a]
EL544 (wt)	wt	*E. coli* MG1655 BW25993 *Δphi80 rph+*	This study	no
**Derivatives of EL544**				
EL421	*J23106-dnaA ΔdnaA*	*ΔgalK::J23106-dnaA ΔpdnaA-dnaA::J23106-dnaA(123 nt)*	This study	no
EL840	*J23106-dnaA ΔdnaA ΔDARS1*	*ΔgalK::J23106-dnaA ΔpdnaA-dnaA::J23106-dnaA(123 nt) ΔDARS1*	This study	no
EL548	*J23106-dnaA ΔdnaA ΔDARS2*	*ΔgalK::J23106-dnaA ΔpdnaA-dnaA::J23106-dnaA(123 nt) ΔDARS2*	This study	no
EL542	*J23106-dnaA ΔdnaA ΔdatA*	*ΔgalK::J23106-dnaA ΔpdnaA-dnaA::J23106-dnaA(123 nt) ΔdatA*	This study	no
EL562	*seqA-venus*	*ΔcobA::seqA-venus-FRTcatFRT*	This study	no
EL2470	*seqA-venus ΔDARS1*	*ΔcobA::seqA-venus-FRTcatFRT ΔDARS1*	This study	no
EL2095	*seqA-venus ΔDARS2*	*ΔcobA::seqA-venus-FRTcatFRT ΔDARS2*	This study	no
EL2097	*seqA-venus ΔdatA*	*ΔcobA::seqA-venus-FRTcatFRT ΔdatA*	This study	no
EL2297	*seqA-venus ΔDARS1 ΔDARS2 ΔdatA*	*ΔcobA::seqA-venus-FRTcatFRT ΔDARS1 ΔDARS2 ΔdatA*	This study	no
EL558	*seqA-venus J23106-dnaA ΔdnaA*	*ΔcobA::seqA-venus-FRTcatFRT ΔgalK::J23106-dnaA ΔpdnaA-dnaA::J23106-dnaA(123 nt)*	This study	no
EL864	*seqA-venus J23106-dnaA ΔdnaA ΔDARS1*	*ΔcobA::seqA-venus-FRTcatFRT ΔgalK::J23106-dnaA ΔpdnaA-dnaA::J23106-dnaA(123 nt) ΔDARS1*	This study	yes
EL586	*seqA-venus J23106-dnaA ΔdnaA ΔDARS2*	*ΔcobA::seqA-venus-FRTcatFRT ΔgalK::J23106-dnaA ΔpdnaA-dnaA::J23106-dnaA(123 nt) ΔDARS2*	This study	yes
EL560	*seqA-venus J23106-dnaA ΔdnaA ΔdatA*	*ΔcobA::seqA-venus-FRTcatFRT ΔgalK::J23106-dnaA ΔpdnaA-dnaA::J23106-dnaA(123 nt) ΔdatA*	This study	yes
EL771	*seqA-venus J23106-dnaA ΔdnaA ΔDARS1 ΔDARS2*	*ΔcobA::seqA-venus-FRTcatFRT ΔgalK::J23106-dnaA ΔpdnaA-dnaA::J23106-dnaA(123 nt) ΔDARS1 ΔDARS2*	This study	no
EL929	*seqA-venus J23106-dnaA ΔdnaA ΔDARS1 ΔdatA*	*ΔcobA::seqA-venus-FRTcatFRT ΔgalK::J23106-dnaA ΔpdnaA-dnaA::J23106-dnaA(123 nt) ΔDARS1 ΔdatA*	This study	no
EL502	*seqA-venus J23106-dnaA ΔdnaA ΔDARS2 ΔdatA*	*ΔcobA::seqA-venus-FRTcatFRT ΔgalK::J23106-dnaA ΔpdnaA-dnaA::J23106-dnaA(123 nt) ΔDARS2 ΔdatA*	This study	no
EL500	*seqA-venus J23106-dnaA ΔdnaA ΔDARS1 ΔDARS2 ΔdatA*	*ΔcobA::seqA-venus-FRTcatFRT ΔgalK::J23106-dnaA ΔpdnaA-dnaA::J23106-dnaA(123 nt) ΔDARS1 ΔDARS2 ΔdatA*	This study	no
EL931	*seqA-venus J23106-dnaA ΔdiaA*	*ΔcobA::seqA-venus-FRTcatFRT ΔgalK::J23106-dnaA ΔpdnaA-dnaA::J23106-dnaA(123 nt) ΔdiaA*	This study	no
EL2128	*seqA-venus J23106-dnaA ΔdiaA ΔDARS2 ΔdatA*	*ΔcobA::seqA-venus-FRTcatFRT ΔgalK::J23106-dnaA ΔpdnaA-dnaA::J23106-dnaA(123 nt) ΔdiaA ΔDARS2 ΔdatA*	This study	no
EL3709	*malI-venus-malO* (34.4 kb from *oriC*)	*intC::P59-malI-mVenusNB-frt ΔgtrA::SpR ΔmalI::tetR yifN::T7p-PLP-BC285::kanR-MalO×12*	This study	no
EL3711	*malI-venus malO* (34.4 kb from *oriC*) *ΔDARS1*	*intC::P59-malI-mVenusNB-frt ΔgtrA::SpR ΔmalI::tetR yifN::T7p-PLP-BC285::kanR-MalO×12 ΔDARS1*	This study	no
EL1548	*seqA-venus plac-dnaA-mcerulean ΔdnaA*	*ΔcobA::seqA-venus-FRTcatFRT ΔlacZYA::dnaA-mcerulean3-FRT ΔpdnaA-dnaA::J23106-dnaA(123 nt)*	This study	yes
EL2822	*seqA-venus plac-dnaA-mcerulean ΔdnaA ΔDARS1 ΔDARS2 ΔdatA*	*ΔcobA::seqA-venus-FRTcatFRT ΔlacZYA::dnaA-mcerulean3-FRT-kanR-FRT ΔpdnaA-dnaA::J23106-dnaA(123 nt) ΔDARS1 ΔDARS2 ΔdatA*	This study	no
EL3242	*seqA-venus J23106-dnaA ΔdnaA tetR-dCas9 /pguide-control*	*ΔcobA::seqA-venus-FRT ΔgalK::J23106-dnaA ΔpdnaA-dnaA::J23106-dnaA(123 nt) tetR-PRoger-PLtetO1-dcas9 /pGuide7-WTO1a*	This study	no
EL3244	*seqA-venus J23106-dnaA ΔdnaA tetR-dCas9 /pguide-pgsA*	*ΔcobA::seqA-venus-FRT ΔgalK::J23106-dnaA ΔpdnaA-dnaA::J23106-dnaA(123 nt) tetR-PRoger-PLtetO1-dcas9 /pGuide7-pgsA*	This study	no
EL3298	*seqA-venus J23106-dnaA ΔdnaA ΔDARS1 ΔDARS2 tetR-dCas9 /pguide-control*	*ΔcobA::seqA-venus-FRT ΔgalK::J23106-dnaA ΔpdnaA-dnaA::J23106-dnaA(123 nt) tetR-PRoger-PLtetO1-dcas9 ΔDARS1 ΔDARS2 /pGuide7-WTO1a*	This study	no
EL3299	*seqA-venus J23106-dnaA ΔdnaA ΔDARS1 ΔDARS2 tetR-dCas9 /pguide-pgsA*	*ΔcobA::seqA-venus-FRT ΔgalK::J23106-dnaA ΔpdnaA-dnaA::J23106-dnaA(123 nt) tetR-PRoger-PLtetO1-dcas9 ΔDARS1 ΔDARS2 /pGuide7-pgsA*	This study	no
EL3308	*seqA-venus J23106-dnaA ΔdnaA ΔDARS1 ΔDARS2 J23106-pgsA*	*ΔcobA::seqA-venus-FRTcatFRT ΔgalK::J23106-dnaA ΔpdnaA-dnaA::J23106-dnaA(123 nt) ΔDARS1 ΔDARS2 ΔintS::J23106-pgsA*	This study	no
EL3408	*seqA-venus Δhda*	*ΔcobA::seqA-venus-FRT lac- Δhda::kanR*	This study	yes
EL2931	*seqA-venus ΔydbL::parS ΔgtrA::mCherry-parB*	*ΔcobA::seqA-venus-FRTcatFRT ΔydbL::parS-FRT-cat-FRT ΔgtrA::p58-mCherry-parB-SpR*	This study	no
EL2990	*ypet-dnaN ΔydbL::parS ΔgtrA::mCherry-parB*	*kan-ypet-dnaN ΔydbL::parS-FRT-cat-FRT ΔgtrA::p58-mCherry-parB-SpR*	This study	no
EL2938	*dnaQ-ypet ΔydbL::parS ΔgtrA::mCherry-parB*	*dnaQ-yfp-FRT-kanR-FRT ΔydbL::parS-FRT-cat-FRT ΔgtrA::p58-mCherry-parB-SpR*	This study	no

[a]The .fastQ files are available at https:/doi.org/10.17044/scilifelab.22139918.v1

Dataset S1 (separate file)*. The number of cells (or equivalent) that were used to create the plots in the main text and supplement. Microscopy configuration numbers, chip size, segmentation algorithm, cell selection criteria, and the number of repeats are also listed.*

Dataset S2 (separate file). *Primers and other synthesized DNA. Includes reference (28).*

Movie S1 (separate file). *Time-lapse of SeqA-Venus fluorescence and phase-contrast images before and after expression of DnaA was turned off. The frame rate was set to 15 frames per second.*

SI References

1. C. E. Castuma, E. Crooke, A. Kornberg, Fluid membranes with acidic domains activate DnaA, the initiator protein of replication in Escherichia coli. *J. Biol. Chem.* **268**, 24665–24668 (1993).

2. K. Sekimizu, A. Kornberg, Cardiolipin activation of dnaA protein, the initiation protein of replication in Escherichia coli. *J. Biol. Chem.* **263**, 7131–7135 (1988).

3. A. S. Gopalakrishnan, Y. C. Chen, M. Temkin, W. Dowhan, Structure and expression of the gene locus encoding the phosphatidylglycerophosphate synthase of Escherichia coli. *J. Biol. Chem.* **261**, 1329–1338 (1986).

4. N. Fingland, *et al.*, Depletion of acidic phospholipids influences chromosomal replication in Escherichia coli. *Microbiologyopen* **1**, 450–466 (2012).

5. W. Xia, W. Dowhan, In vivo evidence for the involvement of anionic phospholipids in initiation of DNA replication in Escherichia coli. *Proc. Natl. Acad. Sci. U. S. A.* **92**, 783–787 (1995).

6. D. Camsund, *et al.*, Time-resolved imaging-based CRISPRi screening. *Nat. Methods* **17**, 86–92 (2020).

7. J. Näsvall, Direct and Inverted Repeat stimulated excision (DIRex): Simple, single-step, and scar-free mutagenesis of bacterial genes. *PLoS One* **12**, e0184126 (2017).

8. G. Charbon, *et al.*, Iron chelation increases the tolerance of Escherichia coli to hyper-replication stress. *Sci. Rep.* **8**, 10550 (2018).

9. G. Charbon, *et al.*, Re-wiring of energy metabolism promotes viability during hyperreplication stress in E. coli. *PLoS Genet.* **13**, e1006590 (2017).

10. G. Charbon, *et al.*, Suppressors of DnaA(ATP) imposed overinitiation in Escherichia coli. *Mol. Microbiol.* **79**, 914–928 (2011).

11. P. P. Cherepanov, W. Wackernagel, Gene disruption in Escherichia coli: TcR and KmR cassettes with the option of Flp-catalyzed excision of the antibiotic-resistance determinant. *Gene* **158**, 9–14 (1995).

12. D. G. Gibson, *et al.*, Enzymatic assembly of DNA molecules up to several hundred kilobases. *Nat. Methods* **6**, 343–345 (2009).

13. H. J. Nielsen, J. R. Ottesen, B. Youngren, S. J. Austin, F. G. Hansen, The Escherichia coli chromosome is organized with the left and right chromosome arms in separate cell halves. *Mol. Microbiol.* **62**, 331–338 (2006).

14. J. Wiktor, *et al.*, RecA finds homologous DNA by reduced dimensionality search. *Nature* **597**, 426–429 (2021).

15. R. Reyes-Lamothe, D. J. Sherratt, M. C. Leake, Stoichiometry and architecture of active DNA replication machinery in Escherichia coli. *Science* **328**, 498–501 (2010).

16. A. Babic, A. B. Lindner, M. Vulic, E. J. Stewart, M. Radman, Direct visualization of horizontal gene transfer. *Science* **319**, 1533–1536 (2008).

17. Ö. Baltekin, A. Boucharin, E. Tano, D. I. Andersson, J. Elf, Antibiotic susceptibility testing in less than 30 min using direct single-cell imaging. *Proceedings of the National Academy of Sciences*, 201708558 (2017).

18. K. Jaqaman, *et al.*, Robust single-particle tracking in live-cell time-lapse sequences. *Nat. Methods* **5**, 695–702 (2008).

19. F. Krueger, F. James, P. Ewels, E. Afyounian, B. Schuster-Boeckler, *FelixKrueger/TrimGalore: v0.6.7 - DOI via Zenodo* (2021) https:/doi.org/10.5281/zenodo.5127899.

20. B. Langmead, S. L. Salzberg, Fast gapped-read alignment with Bowtie 2. *Nat. Methods* **9**, 357–359 (2012).

21. L. Fernández-Coll, *et al.*, The Absence of (p)ppGpp Renders Initiation of Escherichia coli Chromosomal DNA Synthesis Independent of Growth Rates. *MBio* **11** (2020).

22. J. Kenneth, A. Livak, D. Thomas, Schmittgen Analysis of Rela2 tive Gene Expression Data Using Real2Time Quantitative PCR and the 2-ΔΔ C t Method. *Methods* **25**, 4022 (2001).

23. K. Zhou, *et al.*, Novel reference genes for quantifying transcriptional responses of Escherichia coli to protein overexpression by quantitative PCR. *BMC Mol. Biol.* **12**, 18 (2011).

24. K. Miyoshi, Y. Tatsumoto, S. Ozaki, T. Katayama, Negative feedback for DARS2-Fis complex by ATP-DnaA supports the cell cycle-coordinated regulation for chromosome replication. *Nucleic Acids Res.* **49**, 12820–12835 (2021).

25. M. Knopp, *et al.*, A novel type of colistin resistance genes selected from random sequence space. *PLoS Genet.* **17**, e1009227 (2021).

26. Y. Perez-Riverol, *et al.*, The PRIDE database resources in 2022: a hub for mass spectrometry-based proteomics evidences. *Nucleic Acids Res.* **50**, D543–D552 (2022).

27. H. Bremer, G. Churchward, An examination of the Cooper-Helmstetter theory of DNA replication in bacteria and its underlying assumptions. *J. Theor. Biol.* **69**, 645–654 (1977)

28. J. Näsvall, A. Knöppel, D. I. Andersson, Duplication-Insertion Recombineering: a fast and scar-free method for efficient transfer of multiple mutations in bacteria. Nucleic Acids Res. **45**, e33–e33 (2017).
